# Boosting Electrode Performance and Bubble Management via Direct Laser Interference Patterning

**DOI:** 10.1021/acsami.4c20441

**Published:** 2025-01-30

**Authors:** Hannes Rox, Fabian Ränke, Jonathan Mädler, Mateusz M. Marzec, Krystian Sokołowski, Robert Baumann, Homa Hamedi, Xuegeng Yang, Gerd Mutschke, Leon Urbas, Andrés Fabián Lasagni, Kerstin Eckert

**Affiliations:** † Institute of Fluid Dynamics, Helmholtz-Zentrum Dresden-Rossendorf, 01328 Dresden, Germany; ‡ Institute of Process Engineering and Environmental Technology, Technische Universität Dresden, 01062 Dresden, Germany; § Institute of Manufacturing, Technische Universität Dresden, 01062 Dresden, Germany; ∥ Academic Centre for Materials and Nanotechnology, AGH University of Krakow, 30-059 Krakow, Poland; ⊥ Hydrogen Laboratory, School of Engineering, Technische Universität Dresden, 01062 Dresden, Germany; # Fraunhofer IWS, 01277 Dresden, Germany

**Keywords:** alkaline water electrolysis, oxygen evolution reaction, bubble dynamics, direct laser interference patterning, laser-structured electrodes, shadowgraphy

## Abstract

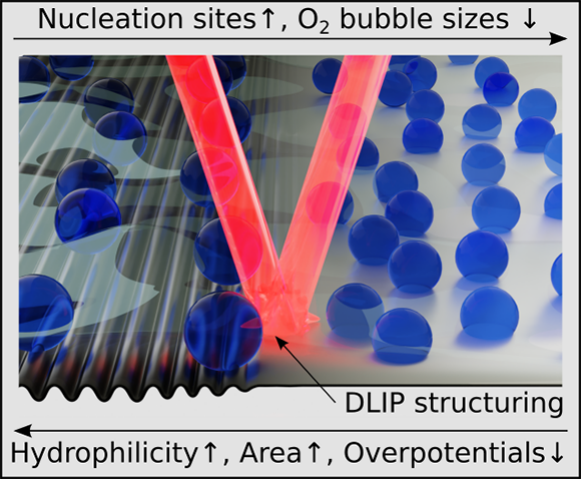

Laser-structuring techniques like Direct Laser Interference Patterning show great potential for optimizing electrodes for water electrolysis. Therefore, a systematic experimental study is performed to analyze the influence of the spatial period and the aspect ratio between spatial period and structure depth on the electrode performance for pure Ni electrodes. Using a statistical design of experiments approach, it is found that the spatial distance between the laser-structures is the decisive processing parameter for the improvement of the electrode performance. Thus, the electrochemically active surface area could be increased by a factor of 12 compared to a nonstructured electrode. For oxygen evolution reaction, a significantly lower onset potential and overpotential (≈ −164 mV at 100 mA cm^–2^) is found. This is explained by the superhydrophilic surface of the laser-structures and the influence of the structured surface on the bubble growth, which leads to a lower number of active nucleation sites and, simultaneously, larger detached bubbles. Combined with the fully wetted electrode surface, this results in reduced electrode blocking and thus, lower ohmic resistance.

## Introduction

Green hydrogen produced by renewable energies using water electrolysis has become a central technology for the transition toward carbon-neutral industry.^1^ Therefore, fossil energies have to be replaced by low or zero-carbon energy sources like solar- or wind-derived electricity to produce hydrogen and replace fossil fuels.^2^ This is particularly necessary in end uses that are difficult to electrify, like heavy transport,^3^ maritime applications^4^ or high-temperature processes like steel^5^ or glass industry.^6^

Besides Proton Exchange Membrane and Solid Oxide Electrolyzers, the most mature technology is still Alkaline Water Electrolysis.^7^ However, large-scale production of green hydrogen still lacks in terms of efficiency, and hence economic competiness. Considerable losses are caused by the evolving hydrogen and oxygen bubbles by increasing ohmic resistances and blocking electrochemically active sites.^8−10^ In addition, the mass transfer and the actual current density is influenced by the electrode coverage.^11−14^ These effects can be reduced by applying external forces,^15−19^ enhancing bubble coalescence,^20,21^ optimizing the electrode’s electrocatalysts,^22^ morphology^15,17,23^ or surface,^17,24,25^ to achieve an optimized bubble nucleation, growth and detachment.

Knowledge of the forces acting on electrogenerated bubble is crucial here. In general, buoyancy,^26^ hydrodynamical^18,26^ and Marangoni,^27−29^ interfacial tension,^17,26,30,31^ contact pressure^31^ and electrical forces^27,32^ act on an individual bubble. In this study, we focus on the interfacial tension force *F*_S_, given by
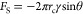
1which acts as an retarding force at the three-phase contact line.^24,27,30^ Here, γ denotes the surface tension. As shown in eq 1, besides the contact radius *r*_c_, the contact angle θ has to be taken into account. In addition, on a rough surface with Cassie–Baxter wetting *F*_S_ is only acting in a fraction of the area (*f*_S_) and thus, the actual interfacial tension force is defined as . This is caused by the discontinuous three-phase contact line due to the nanostructures on the surface.^24,33^ As a result, evolving bubbles are detaching earlier as *F*_S_ is reduced, thus releasing nucleation sites more quickly and finally increasing efficiency.^34^ Furthermore, on a structured surface heterogeneous bubble nucleation can also be enhanced by local surface nanoconfinement, as the critical gas concentration depends on the radius of the nanoparticle serving as nucleation site.^35^

Electroless deposition,^36^ electrodeposition,^37−39^ lithography,^40,41^ UV lithography^23^ and laser techniques^42−46^ have recently been reported to optimize the electrode surface. Laser texturing of surfaces offers several advantages, primarily due to its precise control over the size, shape, and distribution of surface features. Laser-based techniques also enable a reproducible process on an industrial scale,^47^ free from chemical reagents and with minimal waste.^48^

Previous studies have demonstrated the use of laser-based methods to create complex patterns on metallic surfaces, featuring repetitive or periodic arrangements. These structures have been employed to significantly increase electrode surface area, thereby enhancing electrochemical performance of gas evolution reactions.^42−44^ Moreover, when ultrashort laser pulses are applied to metallic surfaces, self-organizing nano- and microstructures are formed, which further increase the electrode surface area and the number of possible nucleation sites. These features, known as Laser-Induced Periodic Surface Structures (LIPSS), arise when materials are irradiated with energy densities near the ablation threshold.^49,50^

For instance, Direct Laser Writing (DLW) has been used to structure nickel electrodes for applications in electrocatalysis and energy storage.^51^ In this context, Rauscher et al. employed a femtosecond pulsed laser to fabricate self-organized conical microstructures on nickel electrodes, achieving a 45% improvement in Hydrogen Evolution Reaction (HER) efficiency.^46^ However, when features with lateral sizes below a few microns are required, DLW faces limitations in throughput and resolution due to the diffraction limit. A promising alternative capable of overcoming these challenges is Direct Laser Interference Patterning (DLIP), which can be seamlessly integrated into automated production lines without significant environmental constraints like UV lithography and offers great flexibility for processing diverse materials.^43,47,48^ This method, when combined with a high-power picosecond laser source, has been used to create periodic line-like patterns with spatial periods of 11 and 25 μm, improving the efficiency of nickel electrodes for HER up to 22%.^42^ Additionally, Ränke et al. employed the DLIP technique in conjunction with a femtosecond pulsed laser to generate highly periodic pillar-like patterns with spatial periods of 3 μm, increasing the Electrochemically Active Surface Area (ECSA) of nickel-based electrodes by almost 10 times and hence achieving a reduction of overpotential of HER by 49%.^52^

As stated above, most reported studies are dealing with HER. However, it is the Oxygen Evolution Reaction (OER) which requires the higher overpotential to overcome of the kinetic barrier, since it is a four electron–proton coupled reaction reaction compared to the two electron-transfer reaction of HER.^53^ For OER the exact reaction mechanism and the ideal electrodes are still being researched.^54^

Therefore, this study systematically investigates the influence of spatial period Λ and structure depth of laser-structured surfaces produced by DLIP on the overall electrode performance during OER. For this purpose, these structures are applied to high-purity Ni as a standard material in alkaline electrolyzers. In addition to surface characterization, the electrochemical performance of the electrodes and the bubble dynamics in terms of detached bubble sizes and number of nucleation sites are analyzed. Using a statistical design of experiment approach, models are developed to further optimize the laser structures.

## Material and Methods

### Design of Experiments

**Figure 1:**
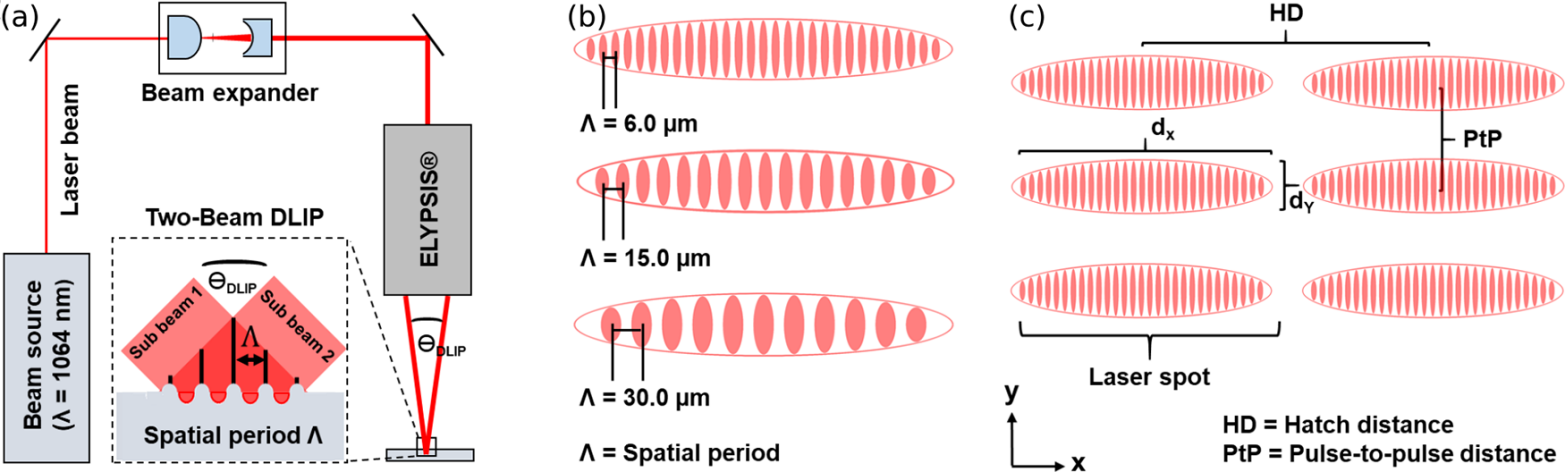
(a) Schematic drawing of the laser texturing showing the two-beam DLIP optical configuration combined with ELIPSYS head. The inset denotes the two overlapping sub-beams producing a line like interference pattern. (b) Resulting interference profiles for spatial periods Λ = 6, 15, and 30 μm. (c) Process strategy for structuring surfaces with elongated laser spots and corresponding structure parameter (spot dimensions *d*_*x*_ = 0.85 mm, *d*_*y*_ = 0.08 mm), pulse-to-pulse distance PtP and hatch distance HD.

In order to study the influence of the laser-structuring on the electrode performance, design of experiments was applied to cover the widest possible range of process parameters and determine the significant structuring parameters.^55^ The design considered three factors: the spatial period Λ, the aspect ratio *AR* between Λ and structure depth (as shown in Figure 1), and the current density *j*. Applying galvanostatic measurement, the influence of these factors on several responses were studied: the quasi-steady state electrode potential *E*_SS_, the bubble size (mode (*d*_m_) and median (*d*_50_) value of the bubble size distribution), and the mean number of active nucleation centers *n̅*_nucl_. Hereby, *E*_SS_ was defined as the time-averaged potential *E̅* of the last 20 s of each measurement. Since, nonlinear effects were expected, a full-factorial design on three levels was chosen to study the relationships. The levels are summarized in Table 1. A detailed measurement plan is shown in Table S1. In order to estimate the measurement noise, the center point was measured independently three times. For better comparison a nonstructured reference electrode was studied as well. Thus, *N* = 3^3^ + 2 + 3 = 32 experiments were scheduled.

**Table 1:** Experimental Parameters of the Design of Experiments with Three Factors and Highlighted Center Point

parameter	range
spatial period Λ (μm)	6, **15**, 30
aspect ratio *AR* (−)	0.33, **0.67**, 1
current density *j* (mA cm^–2^)	10, **31.62**, 100

### Electrode Fabrication

Ni-foils with a thickness of 0.12 mm (GoodFellow, purity 99.95%) were used as substrate for all electrodes. For better comparison, a nonstructured sample (NSE) of the same substrate was cut into the same dimensions of 10 mm × 50 mm.

The laser texturing was performed by employing an optical configuration with two-beam interference optics. The experimental setup consists of a picosecond solid-state laser (Innoslab PX, EdgeWave, Germany) delivering laser pulses with a pulse duration τ of 12 ps and a maximal average laser power of 60 W. The infrared beam (λ = 1064 nm) emitted from the laser source is expanded using a two-lens telescope system and guided into the recently developed optical DLIP head (ELIPSYS SurFuntion GmbH, Germany) that utilizes a Diffractive Optical Element (DOE) to split the incoming main beam into two sub-beams, which later are shaped to elongated lines (see Figure 1a). The introduced optical head enables an impressive depth of focus of ≈10 mm and generates an elliptically shaped laser spot with dimensions (*d*_*y*_ × *d*_*x*_) of 0.08 mm × 0.85 mm in the focal plane. Using this optical setup as shown in Figure 1, considering interference angle θ_DLIP_ as well as the applied laser wavelength λ, the spatial period Λ of the interference pattern can be calculated by
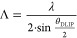
2For this study the spatial period Λ was changed by swapping the DOE within the optical configuration. At a fixed wavelength of λ = 1064 nm, Λ was limited to 6, 10, 15, or 30 μm with the available DOEs. The beam splitting mechanism relies on the optical diffraction grating principle, resulting from a periodic structure atop the DOE surface. Depending on the geometric characteristics of the optical grating, the interference angle θ_DLIP_ of the overlapping beams is modified. For the experimental process three distinct DOEs, each corresponding to spatial periods of 6, 15, and 30 μm, were employed. The movement of the metallic substrates in two orthogonal directions was realized with mechanical stages (Aerotech PRO155-05, USA). The texturing was consistently executed at a fixed repetition rate *f*_rep_ of 10 kHz, using pulse-to-pulse distance *PtP* of 5 μm (see Figure 1c). For the treatment of large areas, the hatch distance *HD*, which is the lateral distance between pulses (see Figure 1c), was set to 300 μm except for Λ = 6 μm, where it was adjusted to 360 μm. This increase in *HD* for the smaller Λ was necessary to prevent partial remelting of the submicro textures due to higher localized thermal loads.

Based on a preliminary study (see Section S2 in the Supporting Information) on the influence of the number of consecutive passes *N* on the resulting structure morphology and Aspect Ratio *AR*, the fabrication of line shaped DLIP features with specific *AR* of 0.33, 0.67, and 1.0 were conducted by adjusting the number of scans from 1 to 27, utilizing distinct single pulse fluences Φ_sp_ ranging from 0.27 to 0.71 J cm^–2^. *AR* is defined to
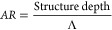
3

### Experimental Methods

**Figure 2:**
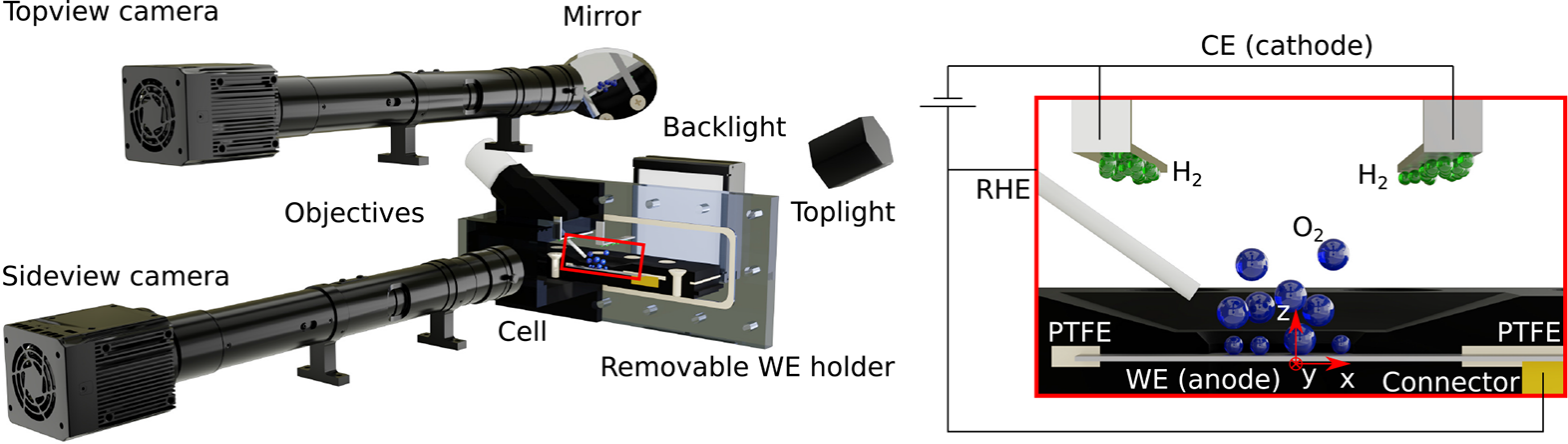
Schematic drawing of the membraneless cell and the optical measurement system used to perform electrochemical measurements and simultaneously study the O_2_ bubble evolution.

A membraneless cell out of PVC (total electrolyte volume *V* ≈ 60 mL) was used to perform all electrochemical experiments, as shown in Figure 2. The working electrode (WE) was mounted on a removable holder and pressed between two sheets of compressible PTFE (PTFE.EXS.100, High-tech-flon, Germany) by 10 M4 screws to ensure proper sealing of the active WE area. The open area of the holder was an elongated hole with a diameter of 2 mm and a length of 10 mm (see Figure S2), which corresponds to an accessible area of the WE of ≈0.23 cm^2^. The counter electrode (CE) consists out of two pieces of Pt-foil (GoodFellow, purity 99.95%) with a total area of  and was placed horizontally at the top of the cell. A reversible hydrogen reference electrode (Mini RHE, Gaskatel, Germany) was placed inside the cell with tip pointing toward the WE, as it is shown in the highlighted section in Figure 2. The design of the cell and the WE holder was an adapted version of the cell setup used in Rox et al.^56^ This cell was used for cyclic voltammetry (CV), linear sweep voltammetry (LSV) and galvanostatic measurements.

All experiments were carried out in a 1 M KOH (Titripur, Merck, Germany) solution under ambient conditions (*T* = 293 K, *p* = 1 bar). Prior to the experiments, the electrolyte was purged with N_2_ for 20 min and pumped into the cell under a N_2_ atmosphere by means of a OB1MK3+ (Elveflow, France). All WEs were cleaned in an ultrasonic bath with Isopropanol for 5 min, rinsed with deionized (DI) water and stored in DI water for at least 48 h to ensure a superhydrophilic surface.^57^ This was proven using a contact angle measurement system (OCA 200, DataPhysics Instruments GmbH, Germany) by applying a water droplet with a volume of 5 μL on the surface, which spreaded directly over the entire structured area, as it is shown in Figure S7 and the examplary video in the Supporting Information. For this purpose, the samples were dried with compressed air before the measurements. Before each measurement performed in the electrochemical cell, the WE was cleaned with ethanol and subsequently rinsed with DI water to remove any remaining contamination before mounting them onto the electrode holder.

#### Characterization of Electrode Surfaces

For evaluating the surface topography of the laser structured samples, White Light Interferometric images (Sensofar S-Neox, Spain) were recorded by employing 50× magnification objective. The surface profiles and average structure depth values were obtained using the SensoMAP Advanced Analysis Software (Sensofar, Spain). In addition, high resolution images of the treated substrates were taken using Scanning Electron Microscopy (SEM) operating at an acceleration voltage of 12 kV (Quattro ESEM, Thermo Fischer Scientific, Germany).

X-ray photoelectron spectroscopy (XPS) measurements were performed to characterize the surface composition. Therefore, monochromatic Al Kα (1486.6 eV) X-rays were focused to a 100 μm spot using a PHI VersaProbeII Scanning XPS system (ULVAC-PHI). The photoelectron takeoff angle was 45° and the pass energy in the analyzer was set to 117.50 eV (0.5 eV step) for survey scans and 46.95 eV (0.1 eV step) to obtain high energy resolution spectra for the C 1s, O 1s, N 1s, P 2p, S 2p and Ni 2p regions. A dual beam charge compensation with 7 eV Ar+ ions and 1 eV electrons was used to maintain a constant sample surface potential regardless of the sample conductivity. All high-resolution XPS spectra were charge referenced to the unfunctionalized, saturated carbon (C–C) C 1s peak at 285.0 eV. The operating pressure in the analytical chamber was less than 3 × 10^–9^ mbar. Deconvolution of spectra was carried out using PHI MultiPak software (v.9.9.3). Spectrum background was subtracted using the Shirley method. Details about the deconvolution and fitting of the high-resolution XPS spectra can be found in Section S8 in the Supporting Information. In addition, for individual electrodes detailed XPS measurements were performed to differentiate between bright and dark areas caused by *HD* of the laser-structuring. Therefore, three bright (b1, b2, b3) and three dark (d1, d2, d3) areas where analyzed on each sample.

#### Characterization of the Electrode Performance

Prior to all electrochemical measurements, each electrode was activated by ensuring a constant open circuit potential (OCP) over 5 min and afterward running 200 cycle voltammograms (CV) at a scan rate of ν = 500 mV s^–1^ from 0.2 to 1 V vs RHE. These CVs were also used to specify the potential region where non-Faradaic currents occur to run series of CVs in a range of ±50 mV around a starting potential within this region. In total 14 different ν (0.02, 0.04, 0.06, 0.08, 0.1, 0.2, 0.3, 0.4, 0.5, 0.6, 0.7, 0.8, 0.9, and 1 V s^–1^) were applied and for each ν five cycles were performed with a 1 min break after each set. For the calculation of the double-layer capacitance *C*_dl_ the last three measurements were taken. Linear sweep voltammetry (LSV) was used to calculate the onset potential *E*_on_ of the OER. A potential range from 0 to 2.5 V vs RHE at a scan rate of 100 mV s^–1^ was selected for this purpose. Finally, galvanostatic measurements at fixed current densities (10, 31.62, and 100 mA cm^–2^) according to Table S1 were performed over a time of *t* = 1 min. Therefore, the applied current was calculated for all electrodes by using the open area of the electrode holder of 0.23142 cm^2^. For all electrochemical measurements a Modulab Xm with a Pstat 1MS/s module (Solartron analytical, Ametek, USA) was used as electrochemical workstation.

#### Characterization of the Bubble Evolution

During the galvanostatic measurements the bubble evolution and detachment were optically investigated from two perspectives (see Figure 2). Therefore, two high-speed cameras (1920 × 760 px, IDT OS-7 S3, USA) were used, each equipped with a precision microimaging lens with a magnification of 2 and a 12.5:1 zoom-module (Optem FUSION, USA). This resulted in a resolution of 867.38 px mm^–1^ and 543.124 px mm^–1^ for sideview and topview, respectively. The depth of field was determined to be 335 μm using a calibration plate. A LED-panel (CCS TH2, Japan) as back illumination completed the shadowgraphy measurement system, while the top view was lit at an angle of ≈60° by a M-LED 3000 plus (ILO electronic, Germany). The field of view for the sideview was adjusted right above the electrode cover, to prevent detached bubbles from dissolving, at a sample rate of 250 Hz. To capture the bubble growth the sample rate for topview images was set to 1000 Hz and the field of view was centered in the middle of the *xy*-plane of the WE surface.

The grayscale images, taken with a 12-bit depth, were analyzed using Python 3.9. The image processing followed the segmentation method introduced in Rox et al.^56^ Thus, a machine-learning based approach was chosen to segment the bubbles in both sideview and topview. Therefore, for each camera perspective a stardist model (v.0.8.5)^58,59^ was trained with a randomly chosen set of 240 and 100 manual labeled images for sideview and topview, respectively. After segmentation of the detached bubbles in the sideview images, all objects were linked using trackpy^60^ and finally, blurred bubbles were eliminated by calculating the size-normalized variance of the image Laplacian (Var(Δ)· *d*_B_). Therefore, all segmented bubbles below the 50% quantile of this metric were excluded from further analysis, as they correspond to the most blurred bubbles.^37,56^ For the topview evaluation, the number of detected bubbles were taken as measure of the active nucleation centers. The stardist model was trained to differentiate between bubbles sitting on the electrode and already detached bubbles by means of the shadow of the inclined illumination. Examples for the image processing steps are shown in Figures S3 and S4. All processed data including all relevant metadata is available at 10.14278/rodare.3064.

#### Multiple Regression Analysis

For the multiple regression analysis, the factors (Λ, *AR* and *j*) were transformed into the space between −1 and 1. Therefore, the transformation rules shown in Table 2 were applied to convert the factors from the real experiment to values between −1 and 1. Afterward, a response surface model with
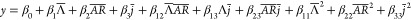
4was fitted for each response *y* (see eq 4), where *y* refers to *E*_SS_, *d*_m_, *d*_50_ and *n̅*_nucl_. Then, the response surface model was reduced applying backward elimination discarding the least relevant factors (threshold: *p*-value >0.05). Thus, at the end a reduced model for each response was derived.

**Table 2:** Transformation Rules for the Multiple Regression Analysis to Scale the Factors from the Real Experiment to Values between −1 and 1

parameter	rule
spatial period Λ	
aspect ratio *AR*	
current density *j*	

Due to high number of scans needed for the electrode with Λ = 30 μm and *AR* = 1.0 and thus, excessive fluence Φ for the given thickness of the substrate, this structure could not be produced reproducibly (see Section S2 in the Supporting Information). Therefore, all measurement points with Λ = 30 μm and *AR* = 1.0 were neglected in this study and *N* was reduced by 3. Thus, all developed models can only provide a first approximation since this corner point of the full-factorial design is missing.

## Results and Discussion

In the following, the electrode surface is first characterized as a function of the structuring process parameters in order to then discuss its influence on the electrochemical performance and bubble dynamics.

### Characterization of the Electrode Structure

As reported in Heinrich et al., by storing the DLIP-structures in DI water the adsorption of organic compounds is limited and thus, the wettability is increased.^57^ The hydrophilicity of the DLIP-structures and their surface chemistry^61^ is supported by the capillary effects of the channels. In comparison to the nonstructured electrode (NSE), with a measured static water contact angle (WCA) of θ_NSE_ = 38.5° ± 2.6°, WCA measurements were not possible for the superhydrophilic DLIP-structures, since the applied droplets spread directly across the entire electrode surface, as it is shown in Figure S7. Thus, for all DLIP-structures it can be stated that θ ≪ θ_NSE_.

**Figure 3:**
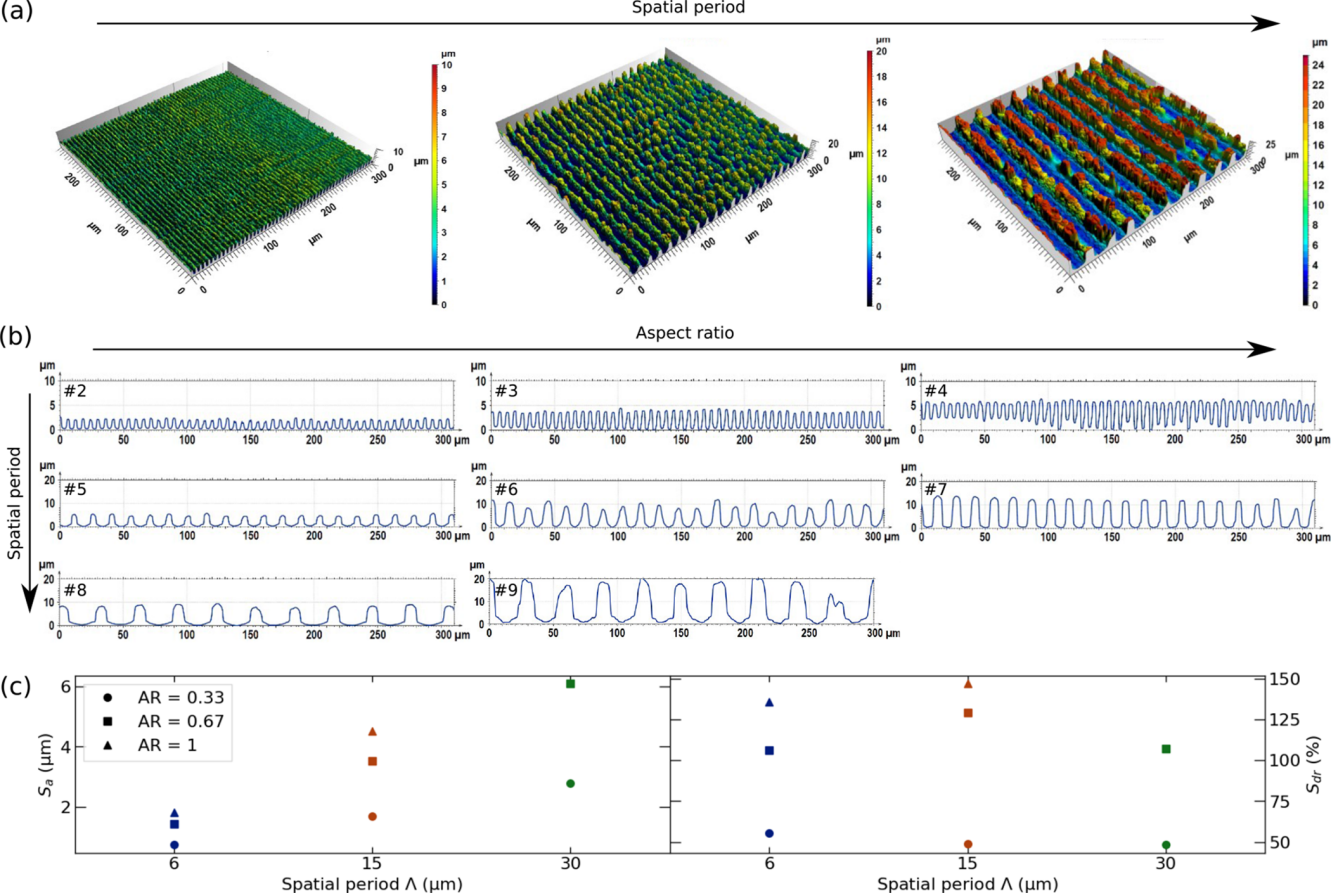
(a) 3D confocal images of DLIP line-like structures with Λ = 6, 15, and 30 μm at *AR* = 0.67. (b) Height profiles of structured Ni-surfaces for all studied electrodes (Λ = 6, 15, and 30 μm and *AR* = 0.33, 0.67 and 1.00). The electrode ID (see Table 3) is provided in the upper left corner. (c) Average surface roughness *S*_a_ and developed interfacial area ratio *S*_dr_ as a function of Λ and *AR*.

Exemplary confocal microscopic images of the DLIP line-like features with an *AR* = 0.67 are shown in Figure 3a revealing that with increasing spatial period Λ the structure regularity is decreasing. In this context, the number of applied scans *N* is the decisive factor, which, as indicated in Figure 3a, was significantly higher for larger structure periods. Generally, the structure formation process was characterized not only by the ablation of the nickel substrate, but also by the redeposition of removed material from the ablation plume.^62^ The amount of redeposited material grows continuously with the increasing number of over scans and therefore had a stronger influence on the regularity of the line-like pattern with Λ = 15 and 30 μm. Furthermore, the redeposition occurred predominantly in the areas of interference minima and did not happen uniformly over the whole surface, leading to the formation of a more irregular DLIP texture. This could also be concluded from the plotted height profiles in Figure 3b, in which, e.g., electrode #9 shows a cutoff peak in the structure.

However, as expected the average surface roughness *S*_a_ followed a linear trend with Λ (see Figure 3c). In contrast, the developed interfacial area ratio *S*_dr_, as a measure of the additional surface area created by the texture compared to the ideal flat substrate, showed a maximum at Λ = 15 μm with an enlargement of ≈150%. The complete confocal images of all DLIP-structures can be found in Figure S5.

**Figure 4:**
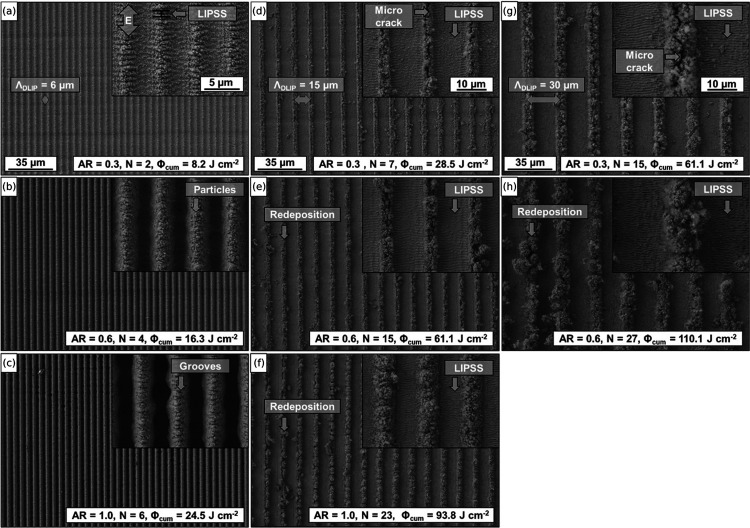
SEM images of DLIP line-like pattern with a spatial period Λ of 6 μm (a–c), 15 μm (d–f) and 30 μm (g, h) fabricated on nickel foils with a single pulse fluence (Φ_sp_) of 0.25 J cm^–2^ and a pulse-to-pulse distance (PtP) of 5 μm. The generated aspect ratio *AR*, the total number of applied passes *N* and cumulated fluence Φ_cum_ are displayed in the corresponding labels. The scale bars in the first row are representative of all columns.

For a more detailed analysis of the surface topographies generated, SEM images were recorded. The line-like DLIP patterns for different *AR* are shown in Figure 4. The patterns displayed in Figure 4a–c depict DLIP structures fabricated with Λ = 6 μm, whereas those in (d–f) exhibit Λ = 15 μm and (g, h) present Λ = 30 μm. The resulting cumulated fluence Φ_cum_, the number of scans *N*, as well as *AR* are given in the labels for each display.

For the samples exhibiting an aspect ratio *AR* = 0.33 the appearance of a homogeneous line-like DLIP pattern decorated with a substructure could be observed for all spatial periods. Upon inspecting the samples structured with Λ = 6 μm (Figure 4a), 15 μm (Figure 4d) and 30 μm (Figure 4g) at a higher magnification (see insets), the presence of a wavy texture could be observed. For the line texture with Λ = 6 μm, all areas of the microstructure shown in Figure 4a were completely covered with the wavy subtextures. In contrast, for the larger structure periods, ripple textures only occurred in the regions corresponding to the interference maxima positions. However, these ripples were oriented perpendicular to the polarization of the applied laser radiation (double arrow E in Figure 4a) and cross the generated DLIP lines pattern at an angle of 90°. The measured periodicity of the ripples Λ_LIPSS_ ranged from 740 to 930 nm, which corresponds to 70–87% of the used laser wavelength (λ = 1064 nm). These characteristics suggested that the substructure can be regarded as LIPSS and further classified as Low-Spatial Frequency LIPSS (LSFL), according to previous studies.^63−65^ Furthermore, the linear textures with Λ equal to 15 and 30 μm, displayed the redeposition of ablated material in the region of minima interference. Apart from this, the formation of microcracks along the structural peaks of DLIP structure became visible for both cases (see Figure 4d,g).

An increase in *AR* to 0.67 led to the homogenization of the DLIP patterns for the 6 μm period, causing the previously visible LSFL substructures to disappear. The redeposition of material in form of nano- and micro particles from the ablation plume was also observed on the structural peaks (Figure 4b).

The resulting DLIP line patterns with an *AR* of 0.67, which are shown in Figure 4e,h, were characterized by the formation of a partially irregular DLIP texture, with increased redeposition of material in the areas of the interference minima. The degree of redeposition was directly dependent on the number of consecutive scans *N*. For instance, processing with a cumulative fluence Φ_cum_ of 110.1 J cm^–2^, resulting from 27 consecutive passes in Figure 4h, led to the continuous growth of redeposition clusters, which started to partially shield the areas of the interference maxima. In general, the homogeneity of the surface structures with an *AR* of 0.67 was observed to steadily decrease with increasing spatial period. Upon closer inspection of the magnified SEM sections for the 15 and 30 μm periods, it was apparent that LIPSS textures were still present in the areas of maxima interference.

The deepest line structures were fabricated for *AR* = 1. The SEM images of the resulting line pattern with Λ = 6 μm continued to show a high degree of homogeneity. Though it became evident that 6 successive passes had influenced the geometric shape of the individual DLIP features. As a result, the borders between interference maxima and minima developed a wavy characteristic and partially even hole-like structures were formed (Figures 4c and S6b).^66^ For Λ = 15 μm in Figure 4f, 23 over scans resulted in a higher amount of redeposited material, leading to an enlargement of line-like DLIP features.

The XPS results in Figures S10–S13 and Table S2 show the relative, chemical composition of the electrode samples. The chemical states and surface concentrations of C, O, and Ni were deconvoluted by fitting high-resolution XPS spectra. No clear tendency of the influence of Λ and *AR* was found for the surface composition. With the exception of the group of aliphatic carbon C–C at a binding energy of 285 eV only little differences were found and thus, the further discussion is focused on electrochemical properties.

### Electrochemical Characterization of the Electrodes

**Figure 5:**
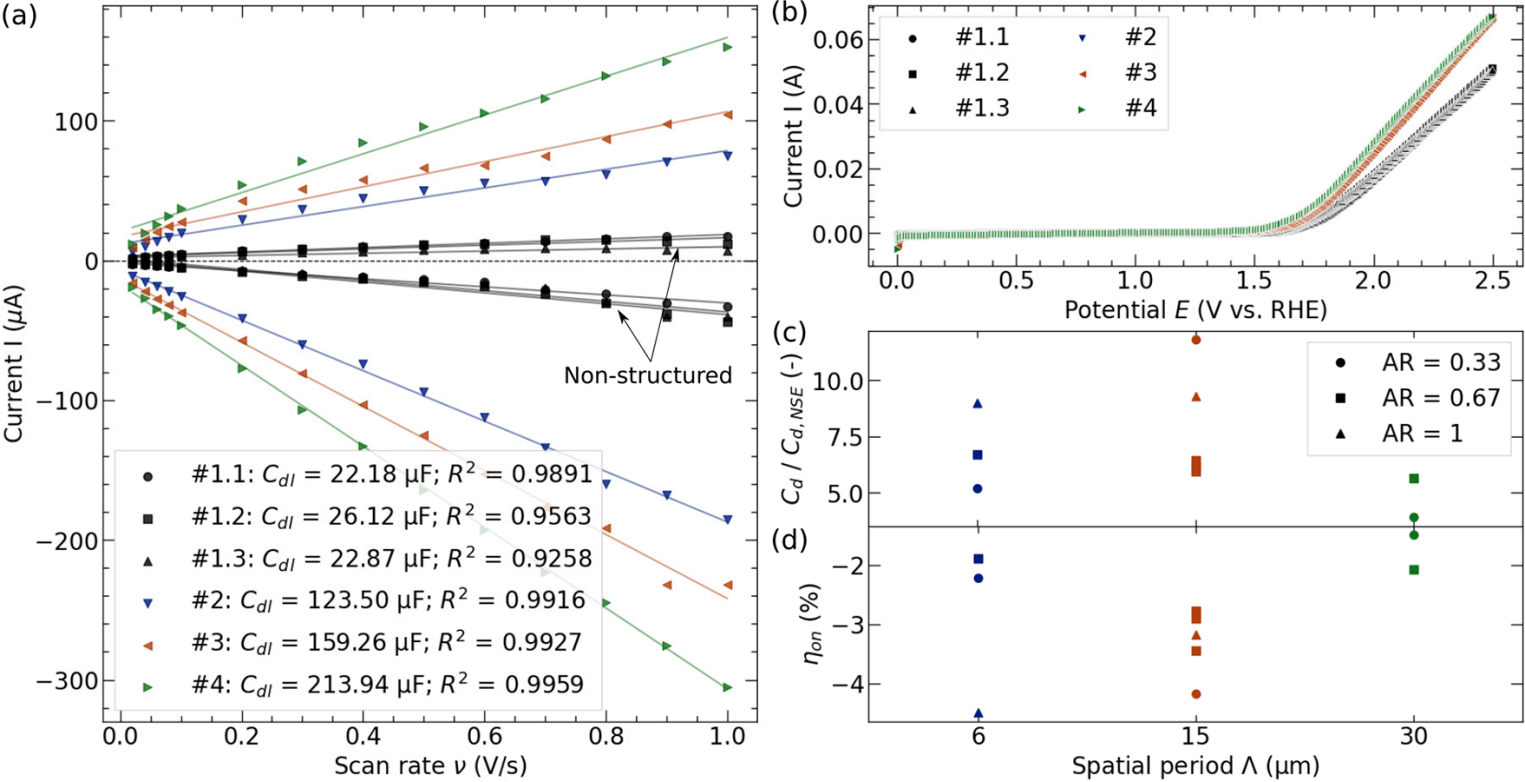
(a) Linear fit of the average anodic and cathodic currents *I* over ν measured during CV and calculated double-layer capacitance *C*_dl_ equal to the slope of the fit for a period length of 6 μm at different aspect ratios. (b) Polarization curves for a period length of 6 μm at different aspect ratios in comparison to NSE. (c) Normalized *C*_dl_ and (d) onset potential η_on_ (see eq 6) for all studied electrodes.

For further characterization of the DLIP-structures, *C*_dl_ as a measure of ECSA was analyzed. *C*_dl_ was calculated as the slope of the linear fits, shown in Figure 5a using
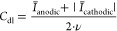
5By running multiple CVs at different ν (see Figure S8), the average currents *I̅* of the last three cycles could be plotted against ν, revealing the linear correlation. In addition to the definition of an unique electrode ID for further discussion of the results, the obtained electrode performance metrics can be found in Table 3.

**Table 3:** Average Surface Roughness *S*_a_ (CM), Double-Layer Capacitance *C*_dl_ (CV), and Onset Potential *E*_on_ (LSV) of All Electrodes – Standard Deviation Was Calculated for Middle and Reference Point of the Design of Experiments

ID	Λ (μm)	*AR* (−)	*S*_a_ (μm)	*C*_dl_ (μF)	*E*_on_ (V)
#1	NSE			23.73 ± 1.72	1.743 ± 0.021
#2	6	0.33	0.749	123.50	1.704
#3	6	0.67	1.43	159.26	1.710
#4	6	1	1.83	213.94	1.664
#5	15	0.33	1.70	280.48	1.670
#6	15	0.67	3.53	146.61 ± 4.70	1.690 ± 0.005
#7	15	1	4.53	220.74	1.687
#8	30	0.33	2.80	92.76	1.717
#9	30	0.67	6.10	133.64	1.707

The shown increase of *C*_dl_ can be attributed to the increase of the developed interfacial area ratio *S*_dr_, since the normalized *C*_dl_ in Figure 5c follows a similar trend like *S*_dr_ in Figure 3c with a maximum at Λ = 15 μm. In addition, when electrode #5 was neglected, the tendency was found that an increasing *AR* leads to a higher *C*_dl_. This also clearly follows the geometric surface enlargement in terms of *S*_a_ and *S*_dr_. However, at low *AR* and Λ = 15 or 30 μm, LSFL were detected in the SEM images, as shown in Figure 4, which contributed to the increased *C*_dl_. In general, it was found that the applied DLIP structuring results in a significant, up to ≈12 × increase of *C*_dl_, which exceeds the increase of *C*_dl_ achieved by laser-structuring in Bernäcker et al. and Baumann et al. However, Bernäcker et al. used short pulse laser-structuring with nonregular laser structures^67^ and Baumann et al. used DLIP structuring at a lower spatial period of Λ = 5.8 μm.^44^

This improvement was also evident in the measured onset potential *E*_on_. For this purpose, the intersection of the non-Faradaic and Faradaic current regions of the recorded polarization curves (see Figure 5b) was defined as *E*_on_, as shown in Figure S9. For better comparability, the efficiency η_on_ was defined with reference to the nonstructured electrode (NSE) as
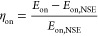
6As a result, it was possible to deduce that the DLIP-structures lead to a reduction of *E*_on_ up to ≈4.5%.

In addition, it was found that the laser-structuring has good reproducibility, as can be seen from the data points at Λ = 15 μm and *AR* = 0.67 in Figure 5c,d or the calculated standard deviations σ for the middle point of the design of experiments in Tables 3 and S3.

For further characterization of the electrode performance, galvanostatic measurements were run over a time of *t* = 60 s at three different current densities of *j* = 10, 31.62, and 100 mA cm^–2^. According to Faraday’s law
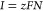
7and assuming equal electrical contact resistances for all measurements, the molar flux of produced O_2_ is constant for all measurements at the same current density.

Since the CE had an area ≈12 × larger than the WE, it was ensured that the HER is not limiting the OER at the WE. Thus, a change in the measured potential *E* of the WE could be directly linked to the anodic overpotential η_anode_ and the ohmic overpotential losses η_Ω_. As a result, a lower measured *E* leads to a decrease of the cell potential *E*_cell_ as an important measure of the overall efficiency of water electrolysis,^68^ which is defined as
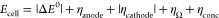
8

As all measurements were carried out in 1 M KOH, the concentration overpotential η_conc_ is negligible.^68^

Similar to eq 6, the efficiency η_SS_ was defined for better comparability of the quasi-steady state potential *E*_SS_:
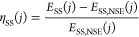
9

**Figure 6:**
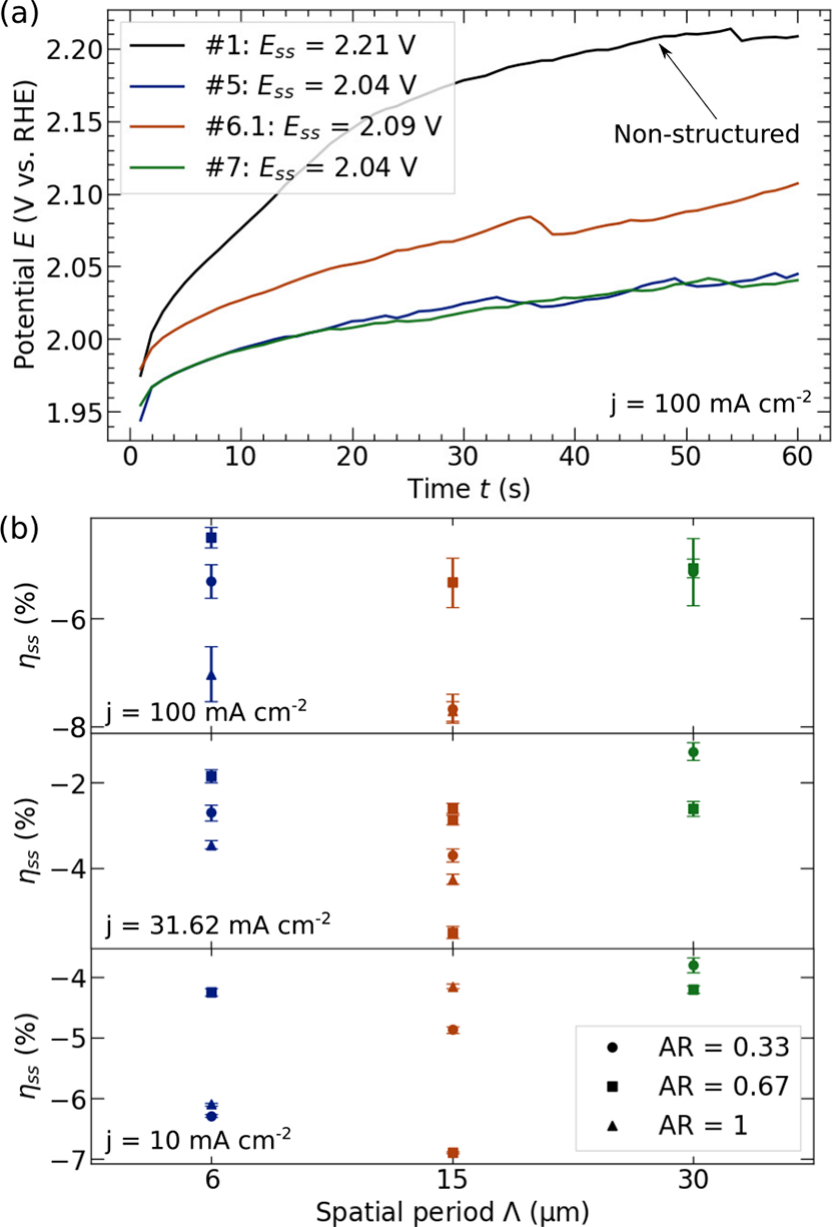
(a) Potential *E* during 60 s galvanostatic measurements for a period length of 6 μm at different aspect ratios. (b) Normalized WE overpotential η_SS_ (see eq 9) calculated from the quasi-steady state electrode potential *E*_SS_ at *j* = 10, 31.62, and 100 mA cm^–2^.

As shown in Figure 6*E*_SS_ was significantly lower for all studied DLIP-structures. The best-performing electrode #7 achieved a ≈164 mV lower *E*_SS_ at *j* = 100 mA cm^–2^ with electrode #5 performing similarly well, as can be seen in Figure 6a. In addition, the calculated measurement noise in Table S3 of σ ≈ 0.024 V served to indicate excellent reproducibility of the DLIP-structuring. It should be emphasized that in Figure 6b η_SS_ follows a similar trend with a maximum at Λ = 15 μm like the normalized *C*_dl_ in Figure 5c. Since no clear influence on η_SS_ can be identified for *AR*, the hypothesis can be made that Λ is the decisive factor for DLIP-structuring.

This was also confirmed on the basis of the multiple regression analysis. The obtained model (*R*^2^ = 0.984) showed a nonsurprising significant influence of *j*. More interesting was the significance of the influence of Λ on *E*_SS_ which is confirmed by the significant (thus not eliminated) linear term Λ̅ and quadratic term for Λ̅^2^ in eq 10. Note that eq 10 is free of physical units due to the transformation explained in Table 2.
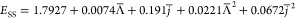
10The shown improvement of η_SS_ of ≈-7.7% can be explained by the smaller effective current density *j*_eff_ due to increased *C*_dl_ and following, ECSA. At a constant molar flux of produced O_2_, this leads to decreased η_Ω_, since more electrode surface is available for the electrode–electrolyte interface. This is also supported by the reduced mass transfer limitations due to the superhydrophilic wetting behavior of the surface.

### Detached Bubble Sizes

**Figure 7:**
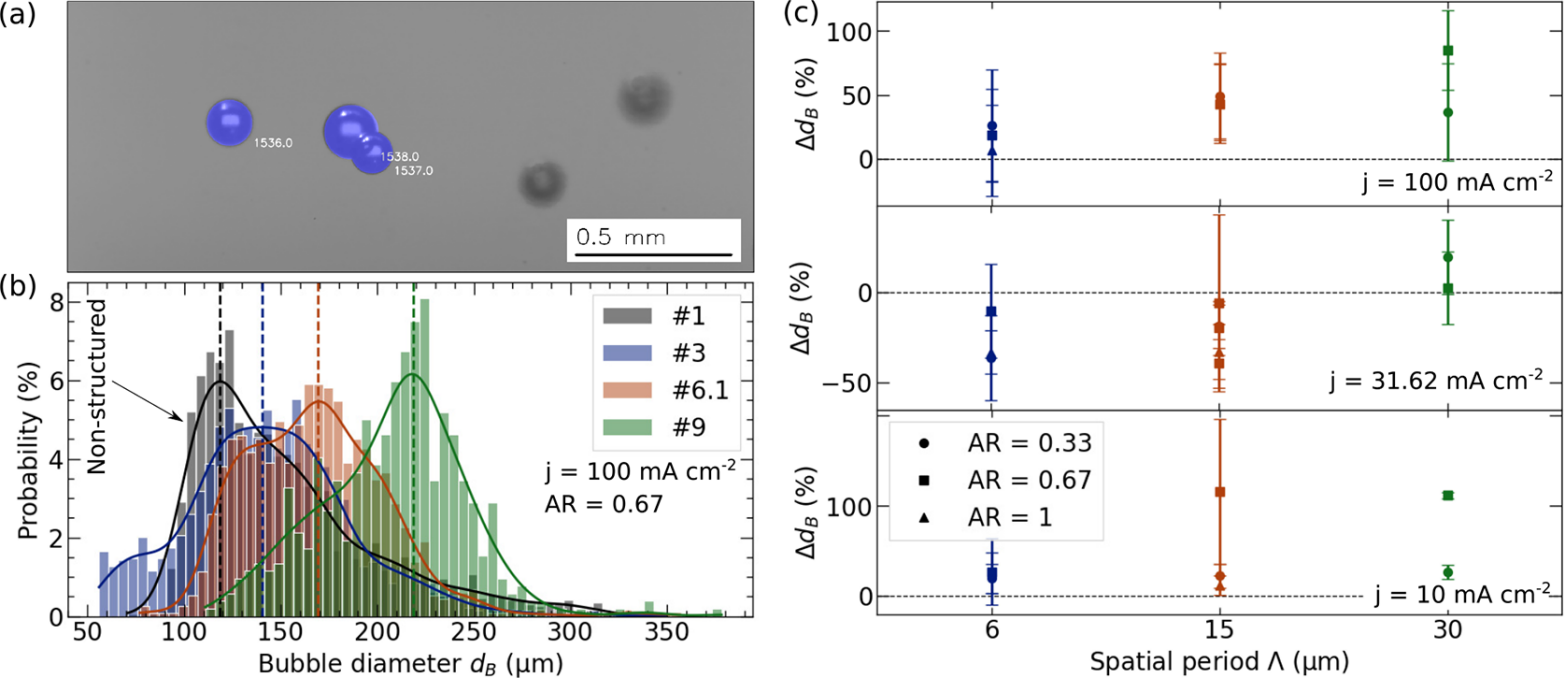
(a) Example sideview image with rising O_2_-bubbles. Those which are inside the focal plane are highlighted by blue color. The numbers represent the unique bubble IDs. (b) Detached bubble size distribution of all periods (Λ(#3) = 6 μm, Λ(#6.1) = 15 μm and Λ(#9) = 30 μm) at a constant *AR* = 0.67 in comparison to NSE (#1) and with mode value *d*_m_ of each bubble size distribution indicated by dash-dotted line. (c) Normalized change of *d*_m_ (see eq 11) at *j* = 10, 31.62, and 100 mA cm^–2^.

For a better understanding of the described improvement of the electrochemical performance of the DLIP-structures, high-speed images of the bubbles were taken during the galvanostatic measurements over a time of *t* = 60 s. This also revealed the influence of the surface structure on the bubble dynamics. An example of these images with segmented bubbles using the machine-learning based image analysis is shown in Figure 7a. It should be pointed out that only sharp bubbles inside the focal plane were included in the further evaluation. As the critical KOH concentration for bubble coalescence of 0.053 M^69^ is clearly exceed with *c*_KOH_ = 1 M used, bubble coalescence is suppressed. In addition, as the field of view was placed right above the electrode cover, it could be assumed that the bubble diameters *d*_B_ in Figure 7b correspond to *d*_B_ at detachment of the electrode surface. Due to the galvanostatic measurement and thus, constant molar flux of produced O_2_, it can be concluded that larger detached bubbles have been growing for a longer time on the electrode surface. With the measured noise of σ = 17.08 μm for *d*_m_ and σ = 19.32 μm for *d*_50_ (see Table S3), the DLIP-structuring also showed good reproducibility in terms of bubble development.

A normalized metric was again defined for the discussion of the detached bubble sizes using the mode value of the bubble size distribution *d*_m_ to
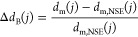
11Due to few active nucleation sites at *j* = 10 mA cm^–2^, high spatial-resolution of the camera (867.38 px mm^–1^) and therefore few to no detected bubbles in the field of view, the evaluation was focused on higher *j*. In addition, as at *j* = 100 mA cm^–2^ the lowest η_SS_ was calculated (see Figure 6b), higher *j* were more relevant for the discussion of the improved electrode performance.

In Figure 7c, it can be seen that at *j* = 100 mA cm^–2^*d*_m_ was larger for all DLIP-structures compared to NSE. This is surprising at first, since smaller bubbles were expected due to the superhydrophilic surfaces of the laser structured electrodes. However, the electrodes used in this study are three-dimensional structures in which the bubbles are more likely to be pinned to the tips of the laser-structures. This will be discussed in more detail in the later section on the active nucleation centers.

It is shown in Figure 7c that an increase in Λ led to a partially linear increase in *d*_m_, with a maximum increase of Δ*d*_B_ ≈ 80% at Λ = 30 μm. However, the influence of *AR* on *d*_B_ had no clear tendency. At *j* = 100 mA cm^–2^, a clear influence of *AR* can only be observed for Λ = 30 μm, assuming larger *d*_B_ with increasing *AR*. However, the exact opposite is seen with a decreased current density of *j* = 31.62 mA cm^–2^. The ambiguity is similarly evident in the obtained models for *d*_m_ (*R*^2^ = 0.812) in eq 12 and *d*_50_ (*R*^2^ = 0.777) in eq 13:
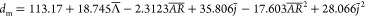
12
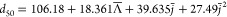
13Interestingly, a significant influence of *AR* could be determined for *d*_m_. Here, *AR* showed a negative influence on *d*_m_, whereas *AR* was eliminated for *d*_50_. Nevertheless, the factor of Λ was again found to be significant and greater for both models. It follows that Λ remains the most important parameter of the DLIP-structuring in terms of electrode performance and detached bubble size. Since the molar flux of produced O_2_ is constant for an applied *j*, it can be concluded that the number of bubbles and thus also the number of nucleation centers *n*_nucl_ was lower.

### Active Nucleation Centers

**Figure 8:**
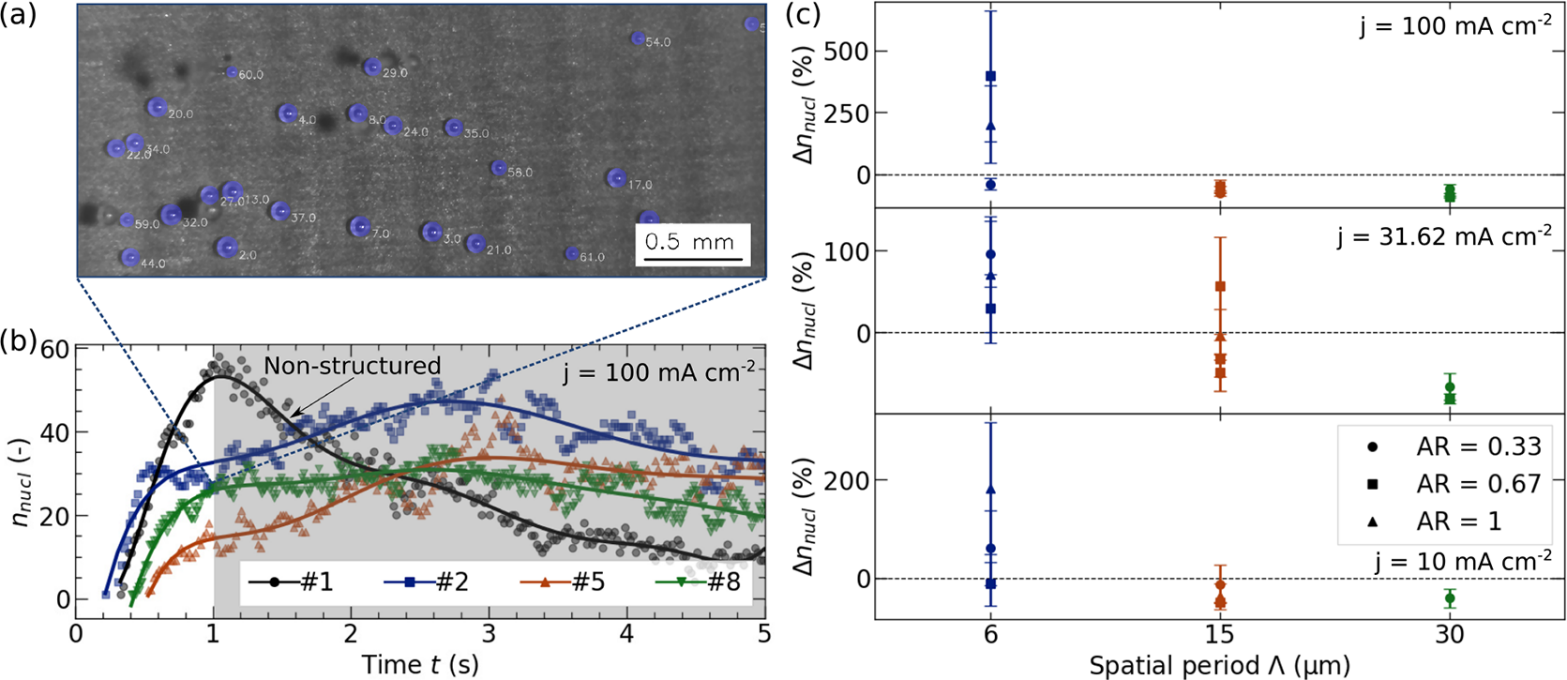
(a) Example topview image with highlighted O_2_-bubbles growing on electrode #2 at *j* = 100 mA cm^–2^ and *t* = 1 s. The numbers represent the unique bubble IDs. (b) Number of O_2_-bubbles during 5 s galvanostatic measurements as a measure of *n*_nucl_ on all periods (Λ(#2) = 6 μm, Λ(#5) = 15 μm and Λ(#8) = 30 μm) at a constant *AR* = 0.33 in comparison to NSE (#1). Only data outside the gray shaded area is included in the further evaluation as rising bubbles are blocking the optical access. (c) Normalized *n*_nucl_ at *j* = 10, 31.62, and 100 mA cm^–2^.

This was confirmed by analysis of the recorded topview images. For that purpose, bubbles sitting on the electrode were segmented using a trained stardist model and taken as a measure for active nucleation centers, as shown in Figure 8a. Due to rising bubbles, the optical access to the electrode surface was limited after a specific time depending on the applied *j*. As shown in Figure 8b, there was a maximum at *t* = 1 s for *n*_nucl_ at *j* = 100 mA cm^–2^. Therefore, depending on *j* only images within the first 4 s, and 1 s, were taken into account to calculate the time-averaged mean of the number of nucleation sites *n̅*_nucl_ at *j* = 31.62 mA cm^–2^ and *j* = 100 mA cm^–2^, respectively. Since few active nucleation sites and small molar flux of produced O_2_, all recorded images were used for *j* = 10 mA cm^–2^. The normalized change in the number of active nucleation sites Δ*n*_nucl_ was then calculated using the following equation:
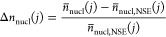
14With the exception of DLIP-structures with Λ = 6 μm, all samples showed a clear decrease in Δ*n*_nucl_ for all applied *j*, as shown in Figure 8c. In addition, an increase in Λ led to a decrease in Δ*n*_nucl_. This could be attributed to the decrease in the number of peaks of the DLIP-structure with increasing Λ, as shown in the height profiles in Figure 3b, where the bubbles are likely to grow.

In combination with the developed model for *n̅*_nucl_ in eq 15 and the results of the bubble size analysis, it could be proven that the DLIP-structures strongly influence the bubble dynamic. Hereby, especially Λ showed a promising approach to tune *d*_B_ and decrease *E*_cell_, as Λ was relevant for all developed models.
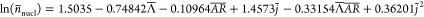
15The shown improvement of the electrode performance in Figure 6b could also be explained by the bubble dynamics. As larger bubbles grew in fewer places on the electrode surface and the line-patterned structure showed superhydrophilic behavior, it was ensured that the surface was largely wetted throughout. Thus, O_2_ could be produced permanently. The dissolved gas was now seemingly collected by the bigger bubbles. This could lead to the conclusion that there were many active catalyst sites but only a small number of nucleation sites. This results in decreased η_Ω_ and following lower *E* were measured.

In addition, coalescence phenomena during the bubble growth on the electrode surface were observed especially at lower Λ, as it is shown in the exemplary videos in the Supporting Information. Bashkatov et al. showed that bubble coalescence leads to earlier bubble departure and thus, can increase the reaction rate.

However, *n*_nucl_ could only be measured during few seconds, whereas *d*_B_ was measured during the full galvanostatic measurements of *t* = 60 s. In addition, as only ≈35% of the entire electrode length of 10 mm was in the field of view in the topview and only ≈22% in the sideview, not all bubbles could be recorded. Especially at low *j*, this could lead to a systematic error if the only active nucleation centers were located outside the field of view. Nevertheless, the measurement noise at the central point of the design of experiments showed only a slight variation (σ = 3.5447, see Table S3) for *n*_nucl_. This further indicates that DLIP results in the formation of a homogeneous structure across the entire electrode. Furthermore, the application of a logarithmic transformation to the response prior to fitting resulted in very good results with *R*^2^ = 0.939. It should be noted that for the model of *n̅*_nucl_, measurements 21 and 26 were excluded from the regression analysis due to the absence of nucleation sites within the field of view.

**Figure 9:**
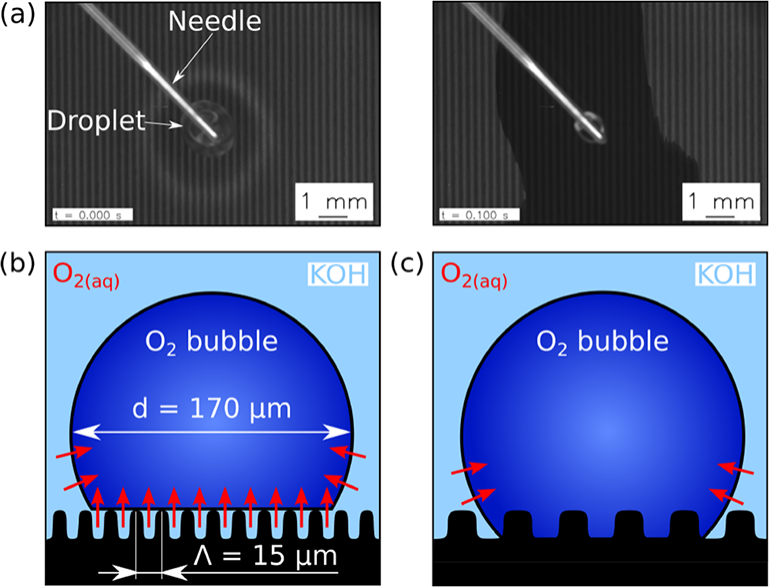
(a) Wetting behavior of laser-structured surface showing superhydrophilic wetting as applied droplet spreads across entire surface. Full time series can be found in Figure S7. (b) Possible nucleation of O_2_ bubble with fully wetted with an assumed bubble size of *d*_*m*_ = 170 μm according to Figure 7 resulting in total of ≈11 covered periods for Λ = 15 μm. (c) Nonwetted electrode surface with increased Λ. As the overaturation of O_2_ is assumed to be larger close to the electrode surface, mass transfer (arrows) is sketched only in the region of the bubble close to the electrode. Qualitative sketches throughout.

Due to limited spatial resolution of the taken topview images, the exact nucleation sites could not be determined. However, as already mentioned above, all laser-structured electrodes showed superhydrophilic wetting behavior. Exemplary images are shown in Figures 9a and S7 indicating the directional spreading of the DI- water along the DLIP line structures. In addition, as all samples were cleaned in a ultrasonic bath and stored for at least 48 h in DI water, it can be assumed that only a negligible amount of gas pockets are present on the electrode surface, where bubbles are most likely to nucleate. It follows that a completely wetted surface is present at the beginning of each measurement. Due to the capillary forces acting in the channels, it can be further assumed that the electrode surface stays in a fully wetted state throughout the measurement. This leads to the assumption that bubble are most likely to be pinned on the tips of the laser-structure as sketched in Figure 9b. Hence, the O_2_ bubble is in a Cassie–Baxter state. This state is the opposite of that of a droplet sitting on a structured surface. For the bubble, electrolyte pockets are confined between the surface and the bubble instead of air pockets. With increasing Λ the capillary forces decrease and thus, it can be assumed that the evolving bubbles covers the whole surface structure (see Figure 9c), and may attain a Wenzel state. However, these are hypothetical considerations that cannot be substantiated at this time.

### Linear Patterned Nucleation Sites

**Figure 10:**
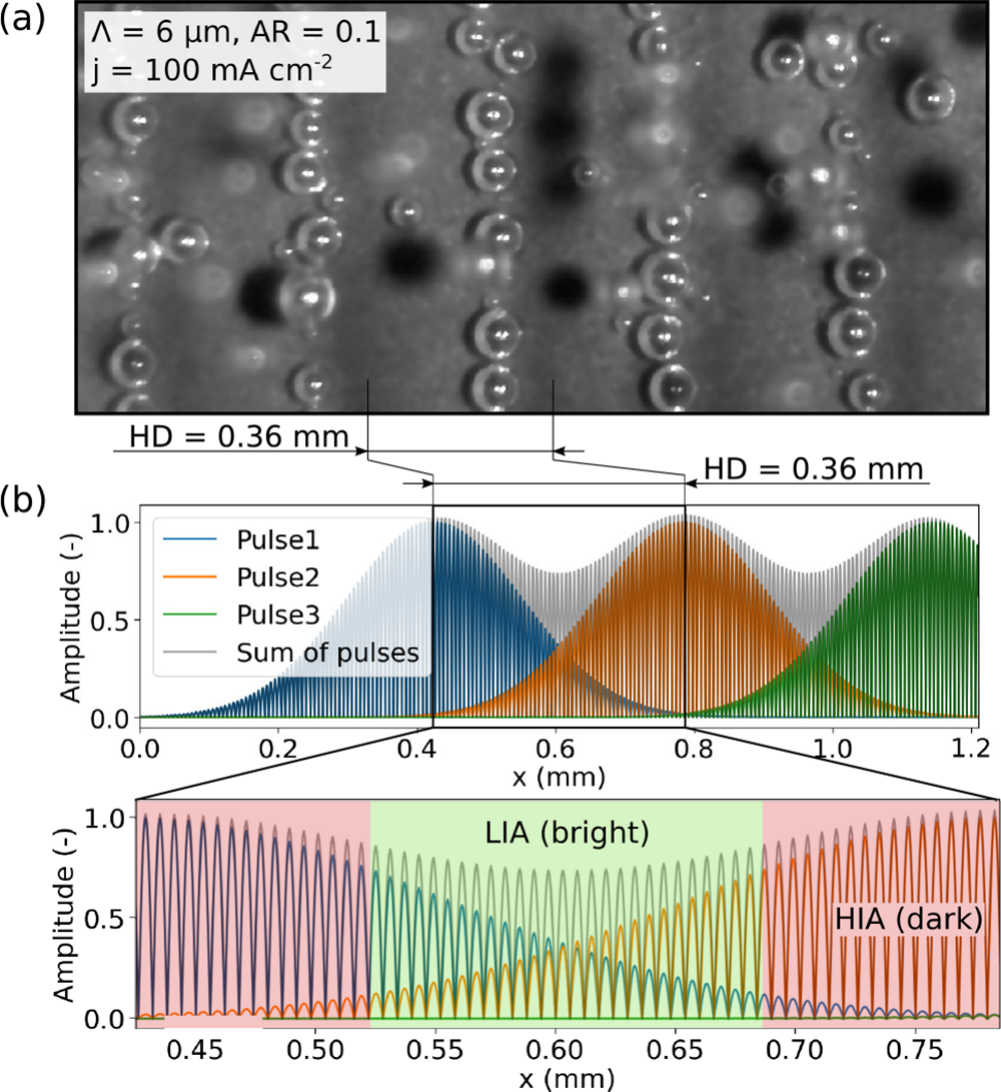
(a) Linear patterned O_2_ bubble nucleation. (b) Superposition of several Gaussian interference patterns similar to the DLIP texturing for the period of 6 μm and the sum of all pulses showing a superimposed change in intensity with a period length equal to the hatch-distance. In addition, the high intensity area (HIA) and low intensity area (LIA) are highlighted in red and green, respectively.

A special phenomena could be observed for the electrodes #3 and #4 with Λ = 6 μm and *AR* = 0.67 and 1, respectively. During OER, the nucleation sites followed a linear pattern, as it is shown in Figure 10a. The period of the visible dark/bright pattern of the electrode surface as well as the distance between the observed lines of active nucleation sites was determined to be equal to *HD* from the DLIP-process. As each laser pulse overlaps in *x*-distance with the previous pulse by *HD*, a superimposed intensity profile with a peak-to-peak distance equal to *HD* is found (see Figure 10b). This results in a darker surface in the region of the high intensity area (HIA) and a brighter surface in low intensity areas (LIA).

Initial assumptions of a changed oxide layer at the electrode surface could be invalidated with the help of XPS measurements, as there was no significant difference in the surface composition between dark and bright areas. This is shown in Table 4, where the chemical states and surface concentrations of O and Ni were deconvoluted by fitting high-resolution XPS spectra (see Figures S14 and S15). Therefore, the average value of the three measurements performed was calculated for both, HIA and LIA.

**Table 4:** Averaged Surface Composition and Standard Deviation (in at %) of the Samples #3 and #4 Determined by Fitting High-Resolution XPS Spectra in the High- (Bright) and Low Intensity Areas (Dark)

element	O			Ni	
BE (eV)	529.5	531.2	532.6	852.3	853.8
groups/ox. state	O–Ni	O–Ni, O=C, O–Si	O–C, −OH, H_2_O abs.	Ni^0^	Ni^2+^
#3 LIA	10.3 ± 0.5	12.7 ± 0.6	12.6 ± 0.9	1.0 ± 0.1	8.6 ± 0.4
#3 HIA	9.8 ± 1.1	13.2 ± 0.2	12.3 ± 0.6	1.1 ± 0.1	8.6 ± 0.6
#4 LIA	4.8 ± 0.3	22.5 ± 0.1	9.1 ± 0.1	0.5 ± 0.0	5.6 ± 0.0
#4 HIA	5.7 ± 0.5	21.1 ± 0.3	9.6 ± 0.1	0.3 ± 0.1	6.2 ± 0.4

Hence, the only significant difference between these areas must lie in the micro- and nanostructure of the electrode surface. This was shown using digital microscopy and SEM images. In LIA (bright) a more shallow profile was detected compared to the HIA (dark). In addition, SEM imaging showed microholes within the maxima region of the interference pattern, which are only present in the higher intensity area (see Figures 4 and S6). Thus, as these microholes serve as cavities for the initial bubble nucleation, a line patterned bubble nucleation with a lateral distance equal to *HD* could be observed. However, due to limited spatial resolution of the topview imaging the exact location of the bubble nucleation can not yet be proven and will be studied in future work.

This effect could be interesting with regard to optimized electrode surfaces and morphologies by defining nucleation sites through DLIP-structuring and optimizing the electrolyte flow at these sites. However, more studies are necessary to understand the ongoing mechanisms and long-term measurement have to be performed to proof that this phenomena does not change over time.

## Conclusions

In summary, the present study investigated the enhancement of electrode performance during the oxygen evolution reaction (OER) by employing Direct Laser Interference Patterning (DLIP) structures on Ni foils. Therefore, the influence of the structure parameters of DLIP, spatial period and aspect ratio, on the double-layer capacitance as a measure of the electrochemically active surface area was investigated. Additionally, the onset potential and overpotential during OER were analyzed. The relation between overpotential and O_2_ bubble dynamics could be studied by determining the bubble size distribution and number of nucleation sites during galvanostatic measurements. For that purpose, the Ni-foils were structured with line-like surface features with Λ = 6, 15, 30 μm and *AR* = 0.33, 0.67, 1.00 using a ps pulsed laser and DLIP. By implementing a statistical design of experiments approach, models were derived from the measurements performed to analyze the significance of the influence of the structure parameters of laser-structuring.

An optimum of the spatial period was found for the double-layer capacitance, and thus the electrochemically active surface area, at Λ = 15 μm, which led to an increase by a factor of 12. In general, all laser-structured electrodes showed a significant enlargement of the electrochemically active surface area. Furthermore, the onset potential of OER could be decreased by ≈4.5%. However, at higher aspect ratios the homogeneity of the line-patterns was decreasing due to increased ablation and thus, a nonlinear trend was observed.

The quasi-steady state potential, as a measure of the electrode overpotential, was decreased by up to ≈164 mV at *j* = 100 mA cm^–2^. This shows the great potential of DLIP-structures for OER. As the bubble sizes were increased and the number of active nucleation sites was decreased, the ohmic resistances could be decreased as large surface areas were wetted throughout. Furthermore, since no significant changes in the surface composition were found for the DLIP-structures, it could be inferred that the increased electrochemically active surface area provided more active catalytic sites. However, the dissolved gas was then collected by bigger bubbles at fewer nucleation sites on the DLIP-structured electrodes.

In general, the spatial period had a big impact on the overpotential and bubble dynamics, while the aspect ratio, and thus the depth of the structure, was not relevant for most models developed multiple regression analysis. Therefore, further studies should focus on the structure size instead of the depth.

It was found that the location of bubble nucleation could be tuned by the evolving laser-induced periodic surface structures. In conclusion, DLIP-structuring offers the possibility to enhance the overall efficiency of OER as well as HER and thus, gas evolving water splitting processes in general, e.g. in alkaline or proton exchange membrane electrolyzers. This enables an improvement of energy storage and can thus contribute to foster the transition toward a carbon-neutral industry. Furthermore, DLIP-structuring might tune the detached bubble sizes to the needs of the periphery. Combined with the eventual possible definition of nucleation centers, this could facilitate the development of novel electrodes and even cell types.

## Data Availability

Data for this article, including electrochemical measurement data, raw images and relevant metadata of the performed experiments are available at RODARE at 10.14278/rodare.3064.

## Supporting Information Available

Additional experimental details, materials, and methods, including comments on the preliminary study on DLIP, deconvolution and fitting of high-resolution XPS spectra, and error analysis of experiments; supporting figures of image processing, electrode surfaces and wetting behavior of electrodes, CVs and LSVs, fitted high-resolution XPS spectra, and plots of the derived models for all studied parameters (PDF)
Wetting behavior of exemplary surfaces (MP4)
Topview at j = 100 mA cm^–2^ and *AR* = 0.67 (MP4)

## Nomenclature

*d*_B_: Bubble diameter, m
*d*_m_: Mode of bubble size distribution, m
*d*_50_: Median of bubble size distribution, m
*d*_*x*,*y*_: Laser spot size, m
*n*_nucl_: Number of nucleation centers, –
*N*: Number of experiments/Number of scans, –
*r*_*c*_: Contact radius, m
*HD*: Hatch distance, m
PtP: Pulse-to-pulse distance, m
*AR*: Aspect ratio between Λ and structure depth, –
ECSA: Electrochemically active surface area, m^2^
*C*_dl_: Double-layer capacitance, F
*t*: Time, s
*E*: Electric potential, V
Δ*E*^0^: Reversible cell potential, V
*E*_cell_: Cell potential, V
*E*_SS_: Quasi-steady state potential, V
*E*_on_: Onset potential, V
*I*: Electric current, A
*j*: Current density, A m^–2^
*j*_eff_: Effective current density, A m^–2^
*V*: Volume, m^3^
*A*_el_: Geometrical electrode area, m^2^
*c*: Electrolyte concentration, mol L^–1^
*F*_S_: Surface tension force, N
*T*: Temperature, K
*p*: Pressure, Pa
*S*_a_: Average surface roughness, m
*S*_dr_: Interfacial area ratio, –
*F*: Faraday constant, C mol^–1^
*Ṅ*: Molar flux, mol s^–1^
*z*: Charge number, –
*R*^2^: Coefficient of determination, –

## Greek Symbols

β: Coefficient for multiple regression analysis, –
γ: Surface tension, N m^–1^
γ: Surface tension, N m^–1^
θ: Contact angle, °
θ_DLIP_: Interference angle, °
Λ: Spatial period length, m
λ: Wavelength, m
σ: Standard deviation, –
τ: Pulse duration, s
ν: Scan rate, V s^–1^
Φ_sp_: Single pulse fluence, J m^–2^
Φ_cum_: Cumulated fluence, J m^–2^
yη_on_: Onset potential efficiency, –
η_SS_: Quasi-steady state efficiency, –
η_anode_: Anodic overpotential, V
η_cathode_: Cathodic overpotential, V
η_Ω_: Ohmic overpotential, V
η_conc_: Concentration overpotential, V
