# Supplementary material for: Boosting Electrode Performance and Bubble Management via Direct Laser Interference Patterning
Source: ACS Appl Mater Interfaces. 2025 Jan 30;17(6):9364–77. doi: 10.1021/acsami.4c20441 (PMC11826882; doi:10.1021/acsami.4c20441)
Supplement: Supplementary file 1 — am4c20441_si_001.pdf [file am4c20441_si_001.pdf]

Supporting information: Boosting electrode performance and bubble management via Direct Laser Interference Patterning

Hannes Rox*[a,b], Fabian Ränke[c], Jonathan Mädler[b], Mateusz M. Marzec[d], Krystian Sokolowski[d], Robert Baumann[c], Homa Hamedi[b], Xuegeng Yang[a], Gerd Mutschke[a], Leon Urbas[b], Andrés Fabián Lasagni[c] and Kerstin Eckert†[a,b,e]

[a]Institute of Fluid Dynamics, Helmholtz-Zentrum Dresden-Rossendorf, Bautzner Landstrasse 400, 01328 Dresden, Germany.
[b]Institute of Process Engineering and Environmental Technology, Technische Universität Dresden, 01062 Dresden, Germany.
[c]Institute of Manufacturing, TU Dresden, 01062 Dresden, Germany.
[d]Academic Centre for Materials and Nanotechnology, AGH University of Krakow, 30-059 Krakow, Poland.
[e]Hydrogen Lab, School of Engineering, Technische Universität Dresden, 01062 Dresden, Germany.

*h.rox@hzdr.de
†k.eckert@hzdr.de

S1 Design of Experiments

Full-factorial design

Table S1: Randomized experiment sequence according to full-factorial design for galvanostatic measurements with dropped experiments for $\Lambda = 30\,\mu m$ and $AR = 1.0$ as this structure could not be manufactured reproducibly

Exp. No.	Λ (µm)	AR (-)	j (mA cm^{-2})
1	6.00	0.33	10.00
2	30.00	0.33	100.00
3	30.00	0.33	31.62
4	6.00	1.00	10.00
5	6.00	1.00	100.00
6	15.00	1.00	31.62
7	15.00	0.67	31.62
8	6.00	0.33	31.62
9	15.00	0.67	100.00
10	30.00	0.67	31.62
11	6.00	0.67	10.00
12	15.00	1.00	10.00
13	15.00	0.33	10.00
14	30.00	0.67	100.00
15	6.00	0.33	100.00
16	15.00	0.33	100.00
17	6.00	1.00	31.62
18	15.00	0.33	31.62
19	15.00	0.67	31.62
20	6.00	0.67	31.62
21	15.00	0.67	10.00
22	6.00	0.67	100.00
23	30.00	0.33	10.00
24	15.00	1.00	100.00
25	15.00	0.67	31.62
26	30.00	0.67	10.00
27	NSE		10.00
28	NSE		31.62
29	NSE		100.00

S2 Preliminary experiments on DLIP

In the first set of experiments, nickel substrates were irradiated using the two-beam DLIP configuration to generate line shaped surface features, exhibiting spatial periods of 6.0, 15.0 and 30.0 µm. As process parameter the number of consecutive passes N were varied from 1 to 45 to evaluate their influence on the resulting structure morphology and aspect ratio AR. For this purpose, the total amount of energy that is used to irradiate a certain area (cumulated

laser fluence Φ_{cum}) has been calculated using Eq. 1 and 2:

$$N_{\text{pulses}} = \frac{d_{\text{y}} \cdot f_{\text{rep}}}{v_{\text{scan}}} \tag{1}$$

$$\Phi_{\text{cum}} = \frac{E_{\text{p}}}{A_{\text{spot}}} \cdot N_{\text{pulses}} \cdot N, \tag{2}$$

where N_{pulses} denotes the number of laser pulses irradiating the same effective area. In this context, the pulse-to-pulse distance of 5 µm corresponds to 16 accumulated laser pulses per area. $A_{\text{spot}} = \pi \cdot d_{\text{x}} \cdot d_{\text{y}}$ describes the area of the interfering laser beams and N the number of consecutive scans. In all experiments, the repetition rate remained constant, resulting in pulse energies E_{p} of 612 µJ, with calculated cumulated laser fluence values Φ_{cum} spanning from 4.1 to 122.3 J cm^{-2}.

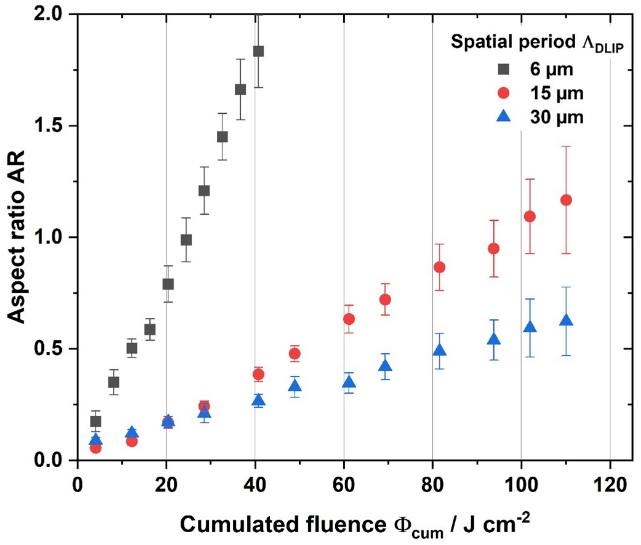

Figure S1: Aspect ratio AR of resulting line-like DLIP structures in dependency of the cumulated laser fluence Φ_{cum} for spatial periods Λ of 6, 15 and 30 µm fabricated with a single pulse fluence Φ_{sp} of 0.25 J cm^{-2} and a pulse-to-pulse distance PtP of 5 µm for different number of scans N.

The generated AR for the single-line experiments (without laser beam hatching) in relation to the applied cumulated fluences are presented in Fig. S1. For this analysis, only the central area of the ablated zone, corresponding to the peak fluence of the Gaussian laser spot, was considered. Across all spatial periods Λ, an increase in cumulative laser fluence resulted in a linear rise of AR for the DLIP line-like features.

The steepest increase in the AR curve was observed for the smallest spatial period of 6 µm, while larger structure periods led to a continuous flattening of the aspect ratio curves. This flattening could be attributed to the enlargement of the maxima regions within the interference profile, resulting in lower ablation rates. Therefore, it can be concluded that higher energy densities (cumulative fluences) are necessary to achieve higher aspect ratios for larger structure periods. For the 6 µm period, the highest AR of 1.8 was achieved, with a greater volume of material vaporized due to the laser-material interaction compared to other structure periods. Maximum aspect ratios of 1.2 and 0.6 were reached for structure periods of 15 µm and 30 µm, respectively.

Based on these results, nickel electrode areas of 25 mm × 10 mm were equipped with line-like DLIP features aiming to generate aspect ratios of 0.33, 0.67 and 1.0 for all mentioned spatial periods. It should be noted that an aspect ratio of 1.0 was not achieved for the structure

period of 30 µm. The reason was the significant bending of the nickel foils caused by high thermal stresses during processing with elevated cumulative energy densities. As a result of this bending, the alignment of the individual laser lines could no longer be maintained, leading to partial destruction of the microstructure.

S3 Working electrode holder

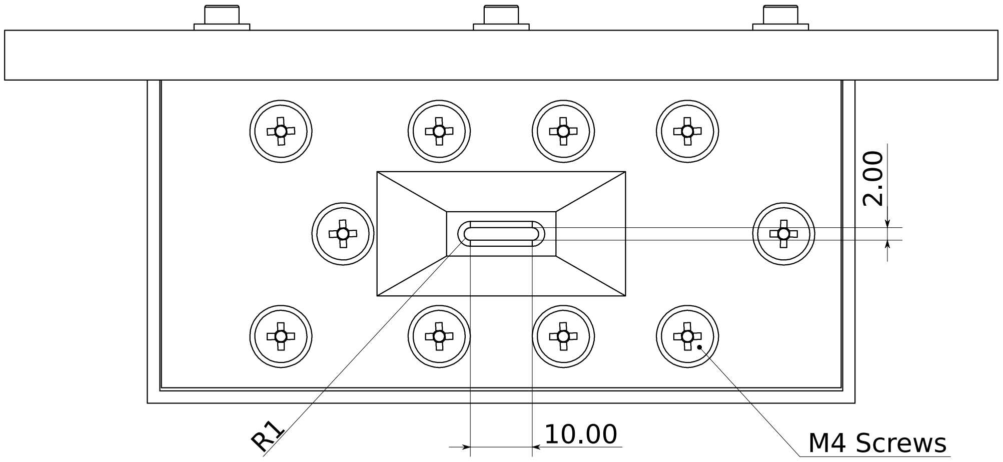

Figure S2: Drawing of the working electrode holder with dimensions of the open area.

S4 Image processing

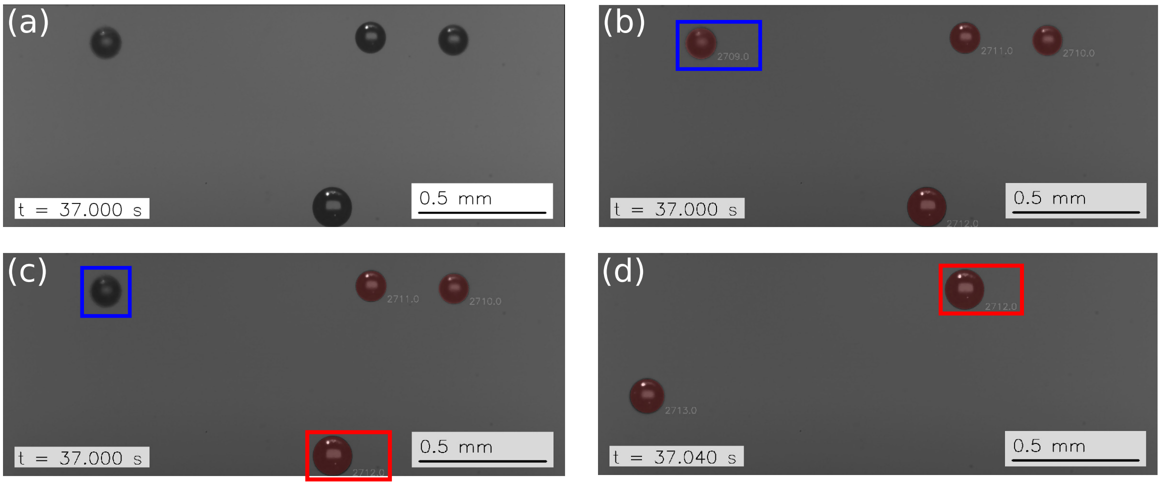

Figure S3: Procedure of image processing of sideview images: (a) Raw image, where (b) all bubble-like objects are segmented and linked (e.g. tracked bubble highlighted in red rectangle in (c-d)) using stardist and trackpy, respectively. Afterwards, by calculating the size-normalized variance of the bubble image Laplacian ($\mathrm{Var}(\Delta) \cdot d_\mathrm{B}$) blurred bubbles, like the highlighted bubble in the blue rectangle can be excluded.

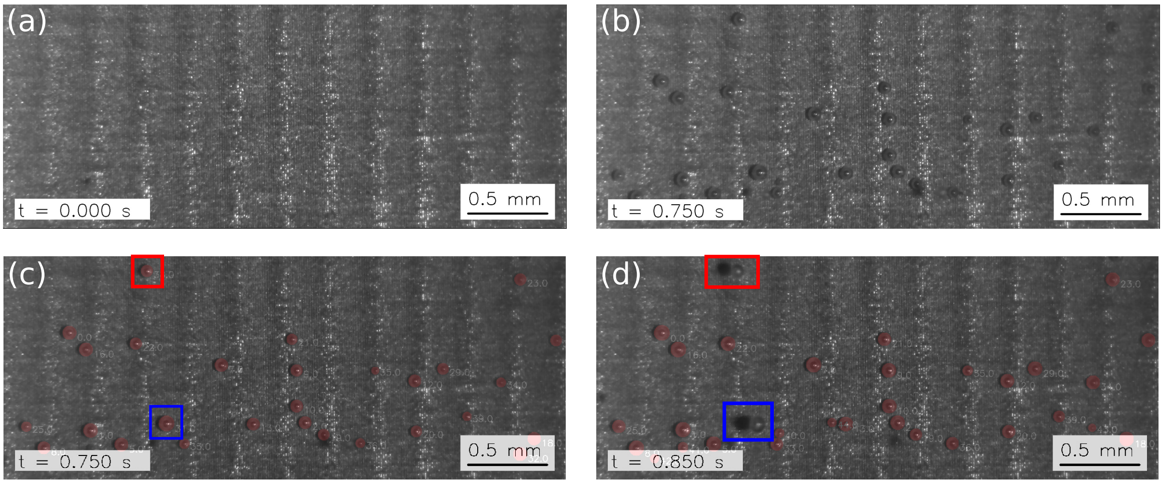

Figure S4: Procedure of image processing of topview images: (a) Clean electrode surface at beginning of experiment and (b) evolving O_2-bubbles after applying j. (c-d) Segmented and linked bubbles sitting on electrode using stardist and trackpy, respectively. The highlighted bubbles show the distinction between bubble sitting on the electrode and detached, rising bubble with a shadow cast on the electrode surface.

S5 Electrode surface

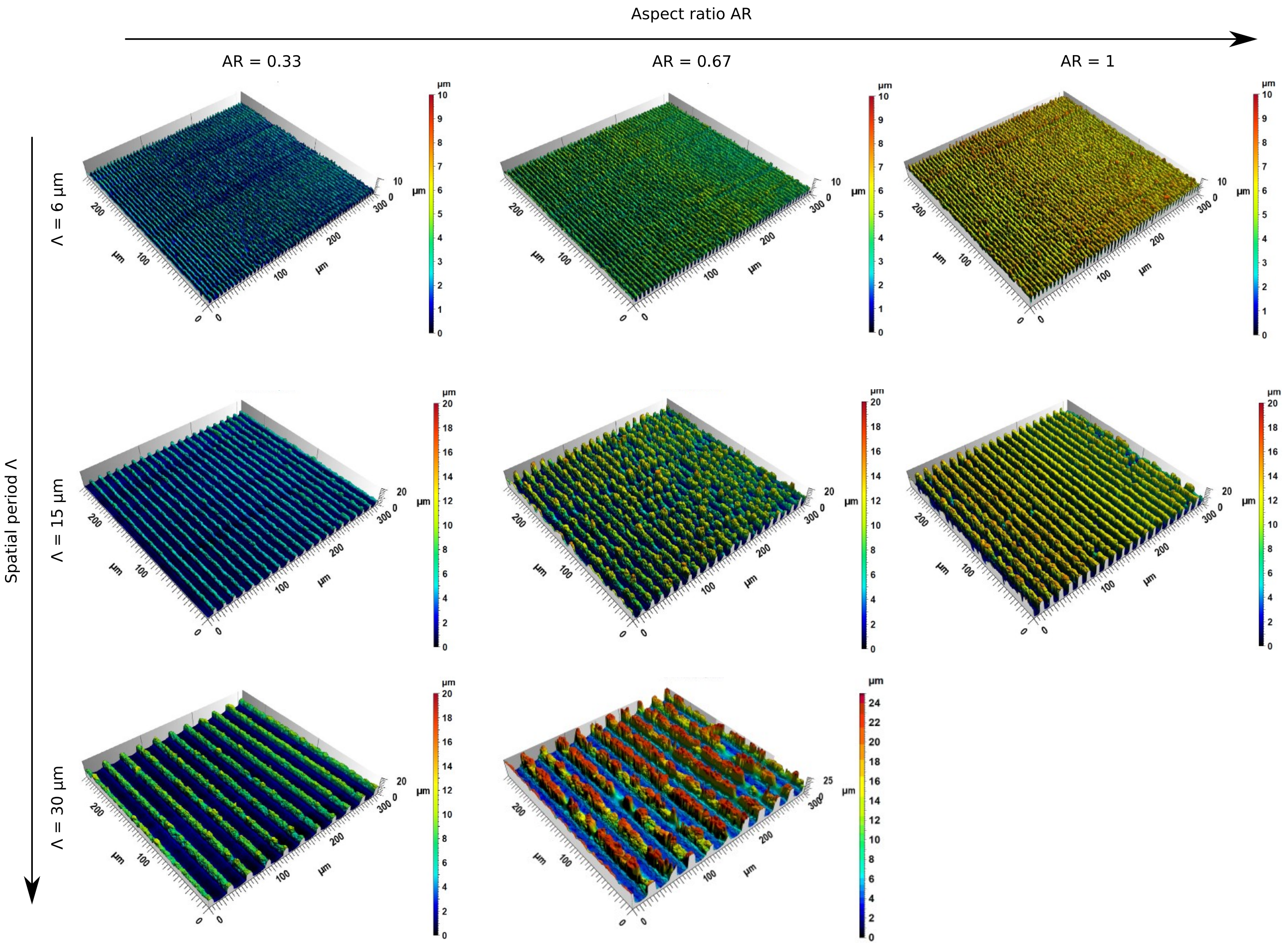

Figure S5: 3D confocal images of all DLIP line-like structures.

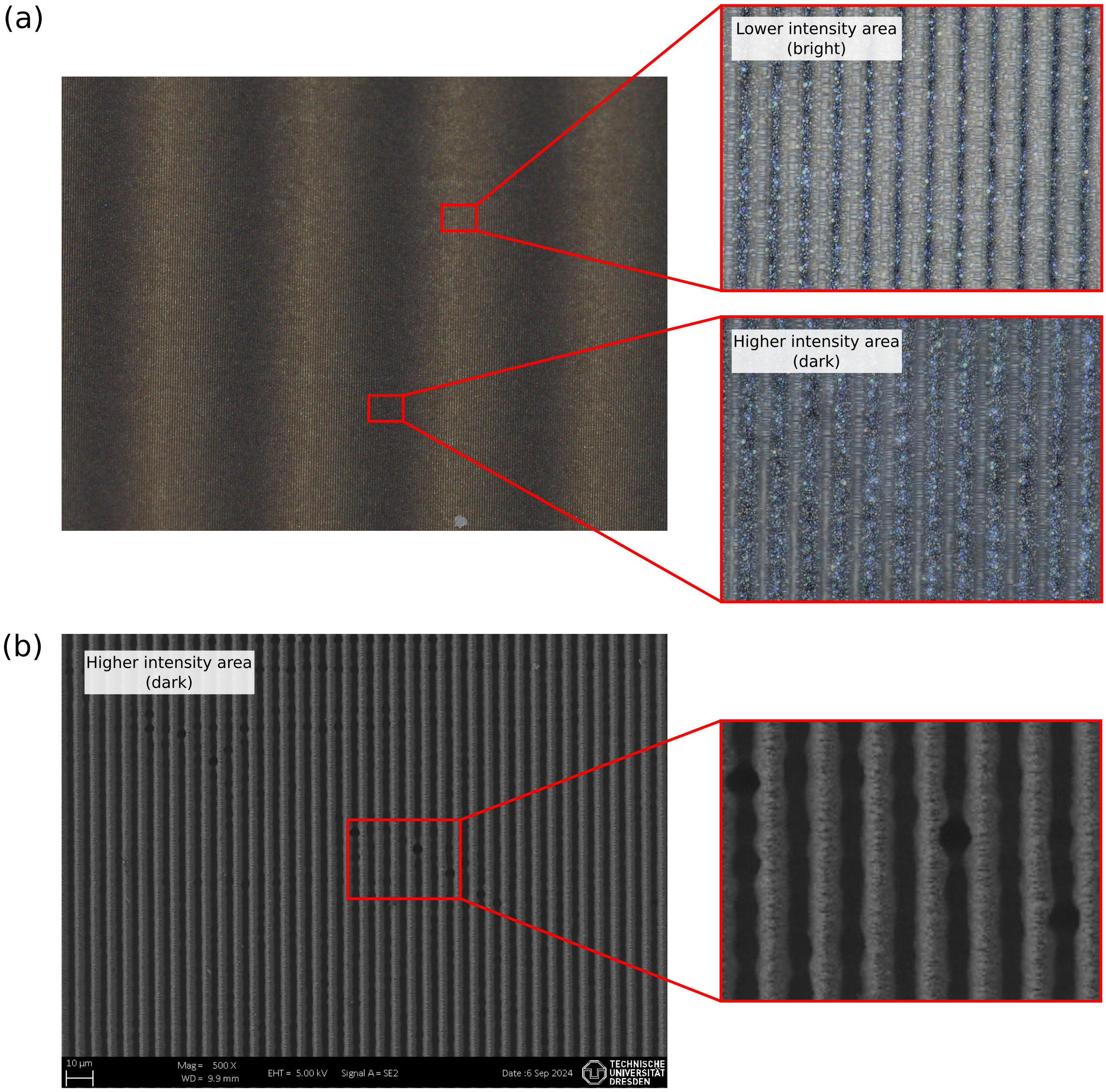

Figure S6: (a) Images of electrode #4 ($\Lambda = 6\,\mu m$ and $AR = 1$) taken with a Keyence VHX Digital Microscope showing a more shallow profile for the brighter area. (b) SEM image of the higher intensity (dark) area of electrode #4 showing microholes in the maxima region of the interference pattern, which are not present in the lower intensity area.

S6 Wetting of electrodes

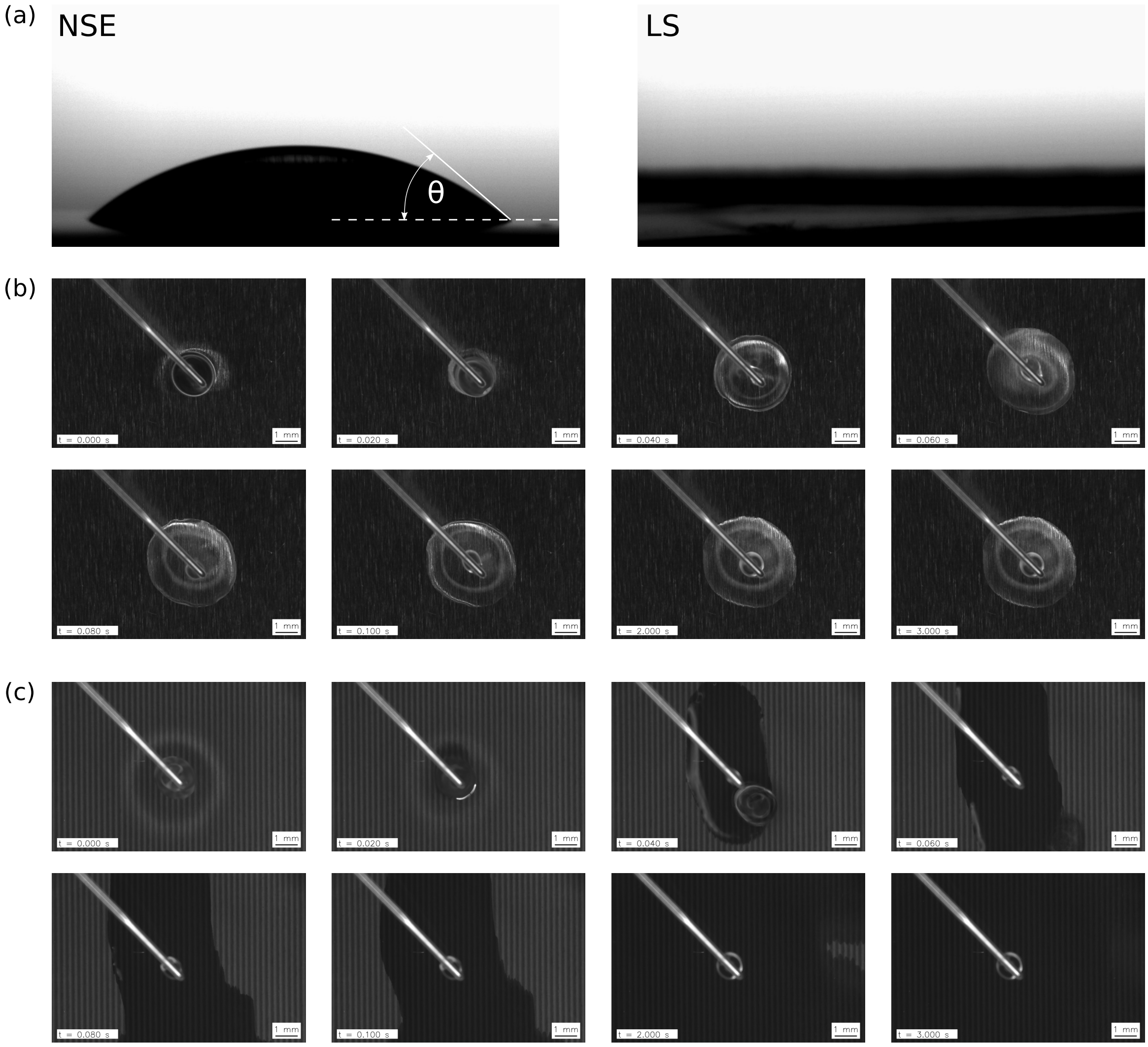

Figure S7: (a) Contact angle measurement of electrodes with highlighted $\theta_{\mathrm{NSE}} \approx 38.5°$ and the non-visible droplet on the DLIP-structured electrode due to superhydrophilic surface. Wetting behaviour of (b) non-structured and (c) laser-structured Ni-foil showing superhydrophilic wetting of laser-structured surface by applying a droplet of ≈ 0.2 ml of DI water on the surface with a 0.4 mm needle and the droplet spreads within less than 3 s across entire surface.

S7 Electrochemical characterization of electrodes

S7.1 Measurement of double-layer capacitance

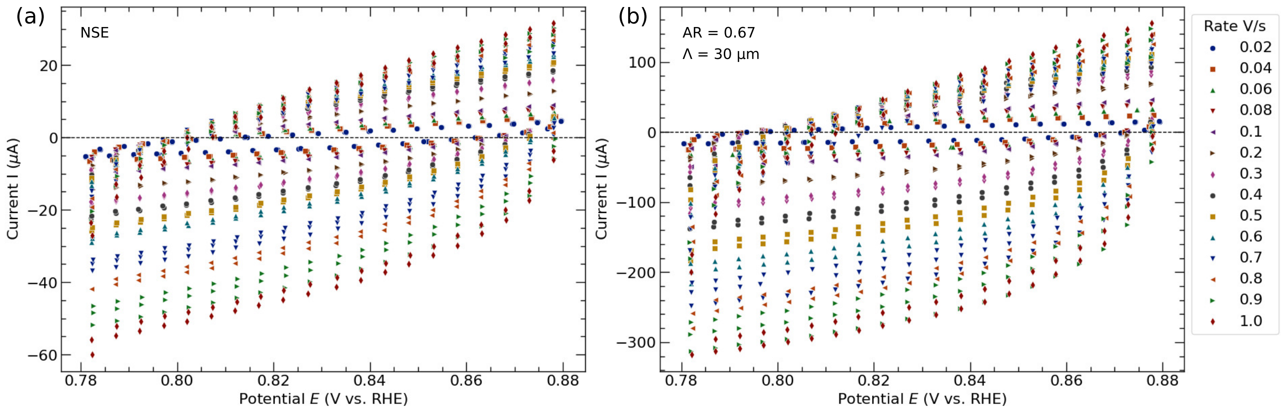

Figure S8: CVs at different scan rates ν for (a) NSE and (b) DLIP-structured electrode.

S7.2 Measurement of onset potential

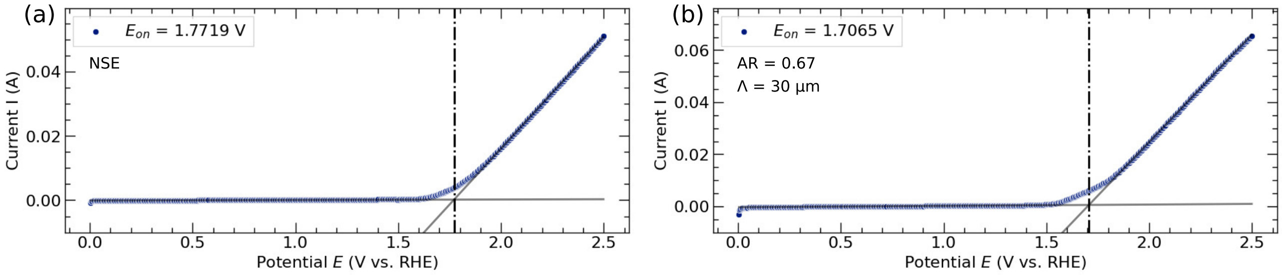

Figure S9: LSVs for (a) NSE and (b) DLIP-structured electrode with fitted tangents to calculate E_{on}.

S8 XPS Spectra

S8.1 Deconvolution and fitting of high-resolution XPS Spectra

For the fitting of the detailed study on the influence of the LIPSS structures (dark/bright pattern) on the electrode surfaces, slightly different values of the binding energy were used for individual spectra. In theses cases, the binding energy used is given in brackets.

The C 1s spectra for all samples were fitted with four components. First line centered at 285.0 eV arise from aliphatic carbon C-C, second line lies at 286.5 eV and indicate presence of C-O and/or C-N bonds, third line centered at 288.2 eV indicate presence of C=O and/or N-C=O bonds[1,2], and fourth line at 289.3 eV indicate presence of O-C=O and or $CO_3{}^{2-}$ type compounds[1].

The N 1s spectra were fitted with up to three lines: first centered at 398.3 eV indicate presence of N=C type bonds, second line at 400.1 eV originates from central three-coordinated nitrogen $N\text{-}C_3$ and/or amine type groups and third line positioned at 402.6 eV which comes from NH_4^+ type ions presence[1,3]. For the LIPSS study only a single line centered at 400.0 eV was used for fitting the N 1s spectra, indicating the presence of N-C=O and/or C-NH type groups[1,3].

The O 1s spectra are similar for all samples and were fitted using three lines, with first line centered at 529.8 eV (529.5 eV) which indicates presence of metal oxide (O-Ni), second line at 531.5 eV (531.2 eV) indicates presence of defective oxygen in metal oxides and/or O=C and/or O-Si type bonds and/or $CO_3{}^{2-}$ groups, and the last line found at 532.2 eV (532.6 eV) which can originate either from O-H and/or C-O type bonds and/or adsorbed H_2O[1,4,5].

The P 2p spectra were fitted with doublet structure ($p_{3/2} - p_{1/2}$ doublet separation equals 0.84 eV) with main $2p_{3/2}$ line centered at 133.2 eV which indicates presence of P^{5+} oxidation state like in PO_4^{3-}[6].

For the LIPSS study instead of the P 2p spectra, the Si 2p spectra show two doublet structures (doublet separation $p_{3/2} - p_{1/2}$ equals 0.6 eV) with first $2p_{3/2}$ line centered at 102.0 eV which indicate presence of C-Si-O type bonds like in silicones/siloxanes[6] and second $2p_{3/2}$ line centered at 103.7 eV which indicate presence of silica type compounds like in e.g. SiO_2[5,6].

The S 2p spectra were fitted with doublet structure ($p_{3/2} - p_{1/2}$ doublet separation equals 1.16 eV) with main $2p_{3/2}$ line centered at 168.3 eV which indicate presence of $SO_3{}^{2-}$ ions[6,7].

The spectra collected at Ni $2p_{3/2}$ region are similar for all samples where nickel was detected. Each spectrum was fitted with up to six lines. First asymmetric line centered at 852.3 eV indicate presence of metallic nickel whereas second line found at 853.8 eV indicate the Ni^{2+} in nickel oxide NiO and/or hydroxide[8–10]. The four lines within energy range of 855 – 866 eV are due to the multiplet splitting phenomena.

S8.2 XPS Spectra and surface composition

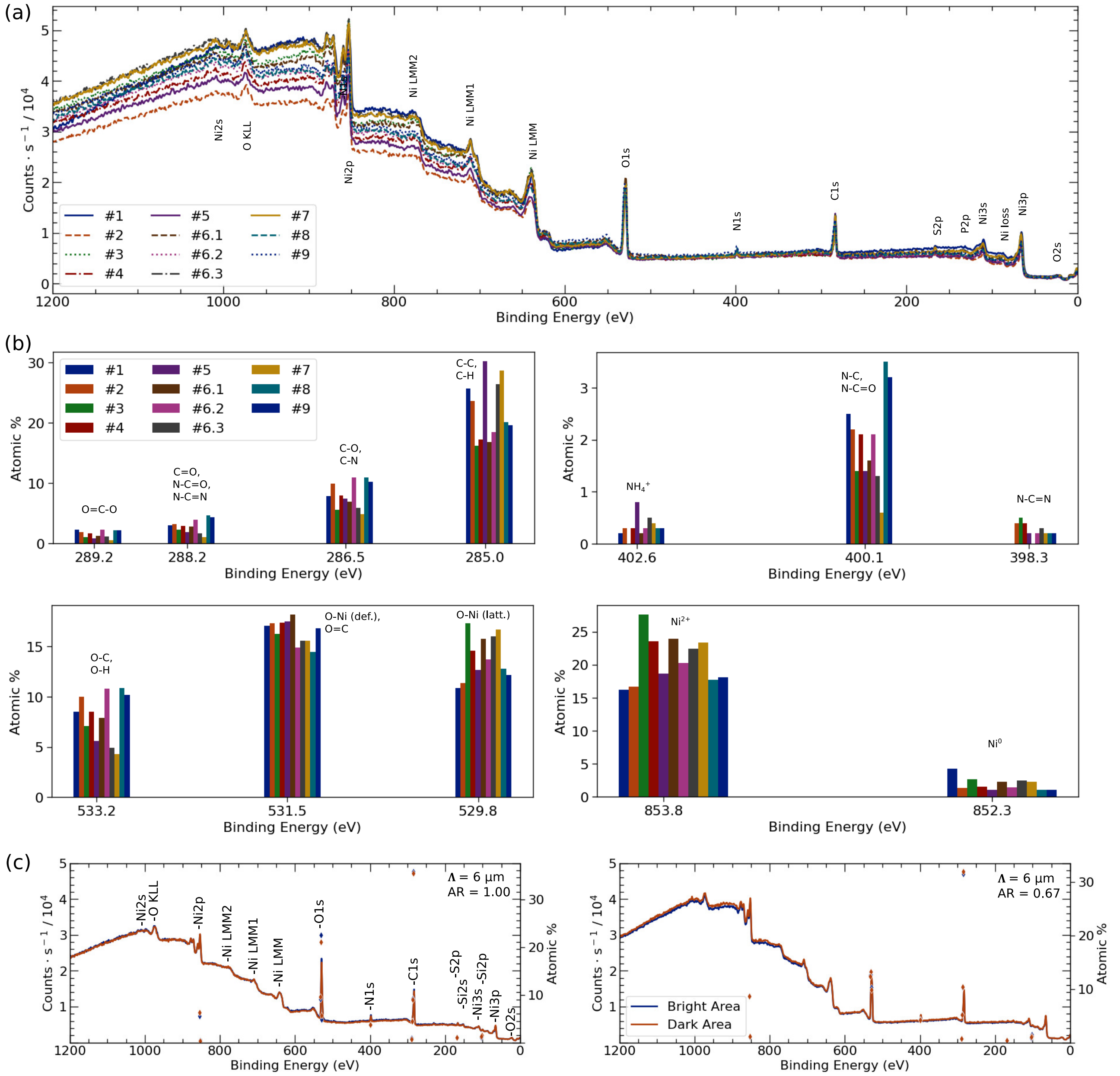

Figure S10: (a) Survey spectra of XPS measurements and (b) surface composition for the Elements C, N, O and Ni for all studied electrodes. (c) Survey spectra of XPS measurements and surface composition inside and outside of *HD* (dark/bright pattern, see Fig. S6) of the two electrodes for which linear patterned bubble nucleation could be observed.

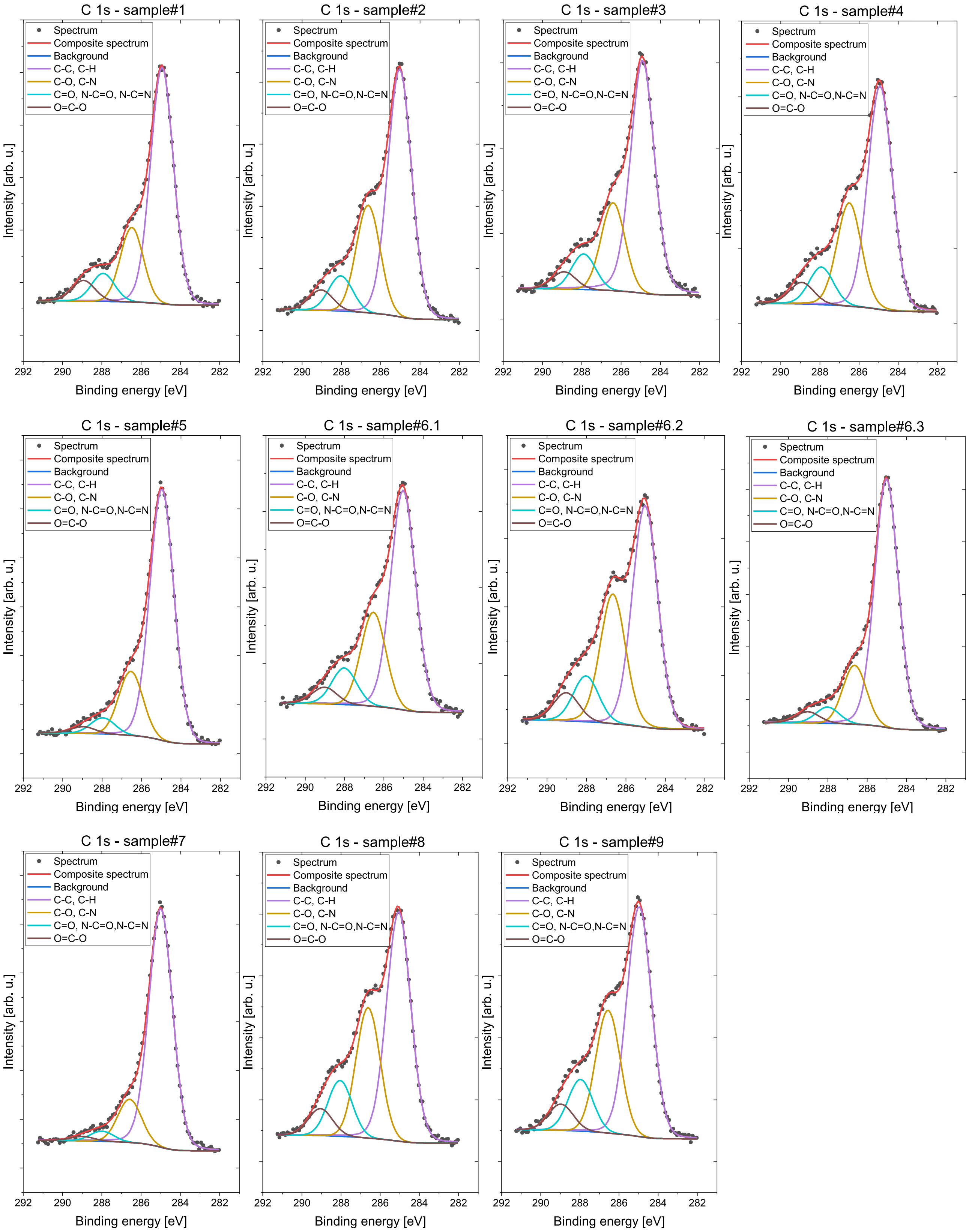

Figure S11: High resolution C 1s spectra of XPS measurements for all studied electrodes.

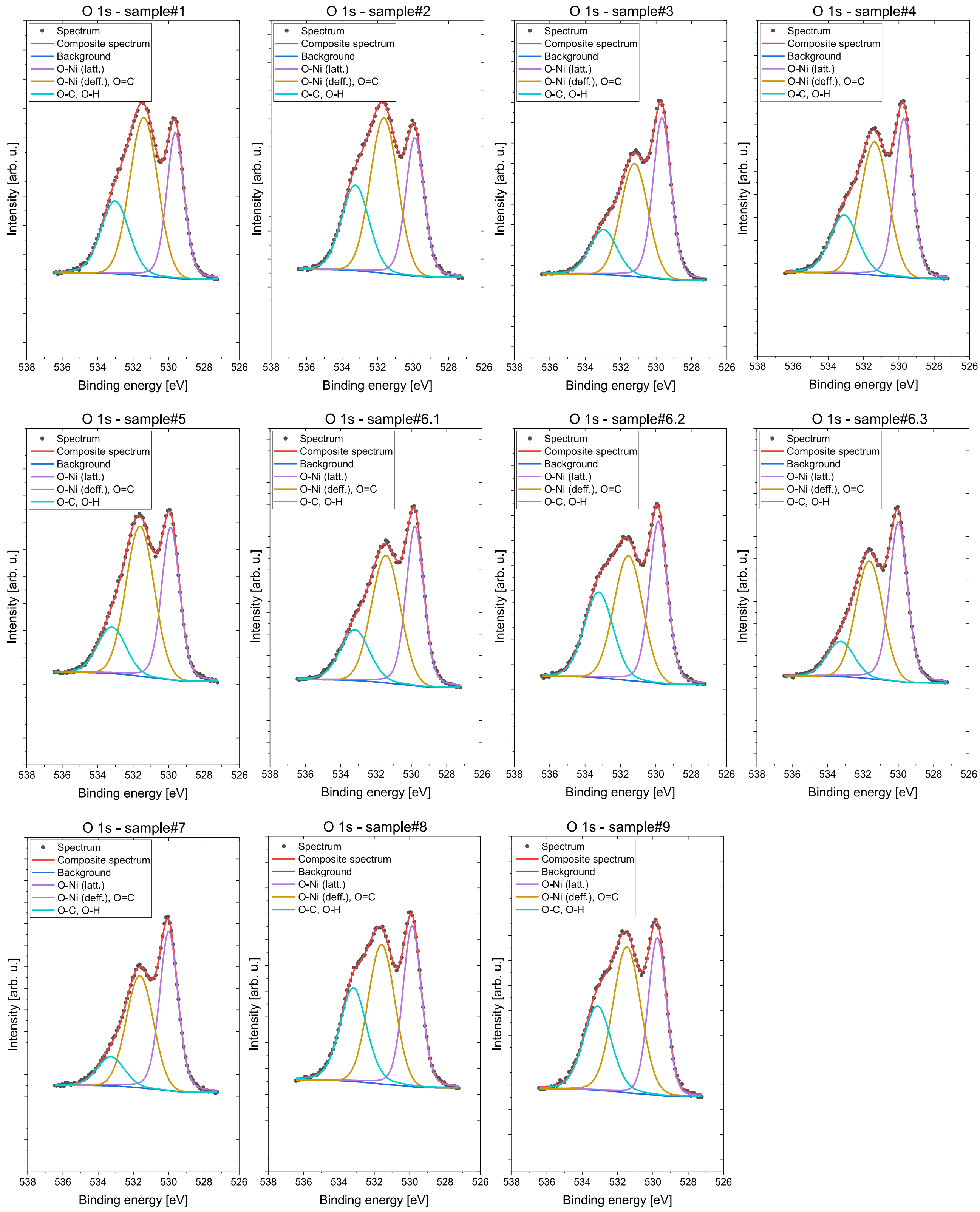

Figure S12: High resolution O 1s spectra of XPS measurements for all studied electrodes.

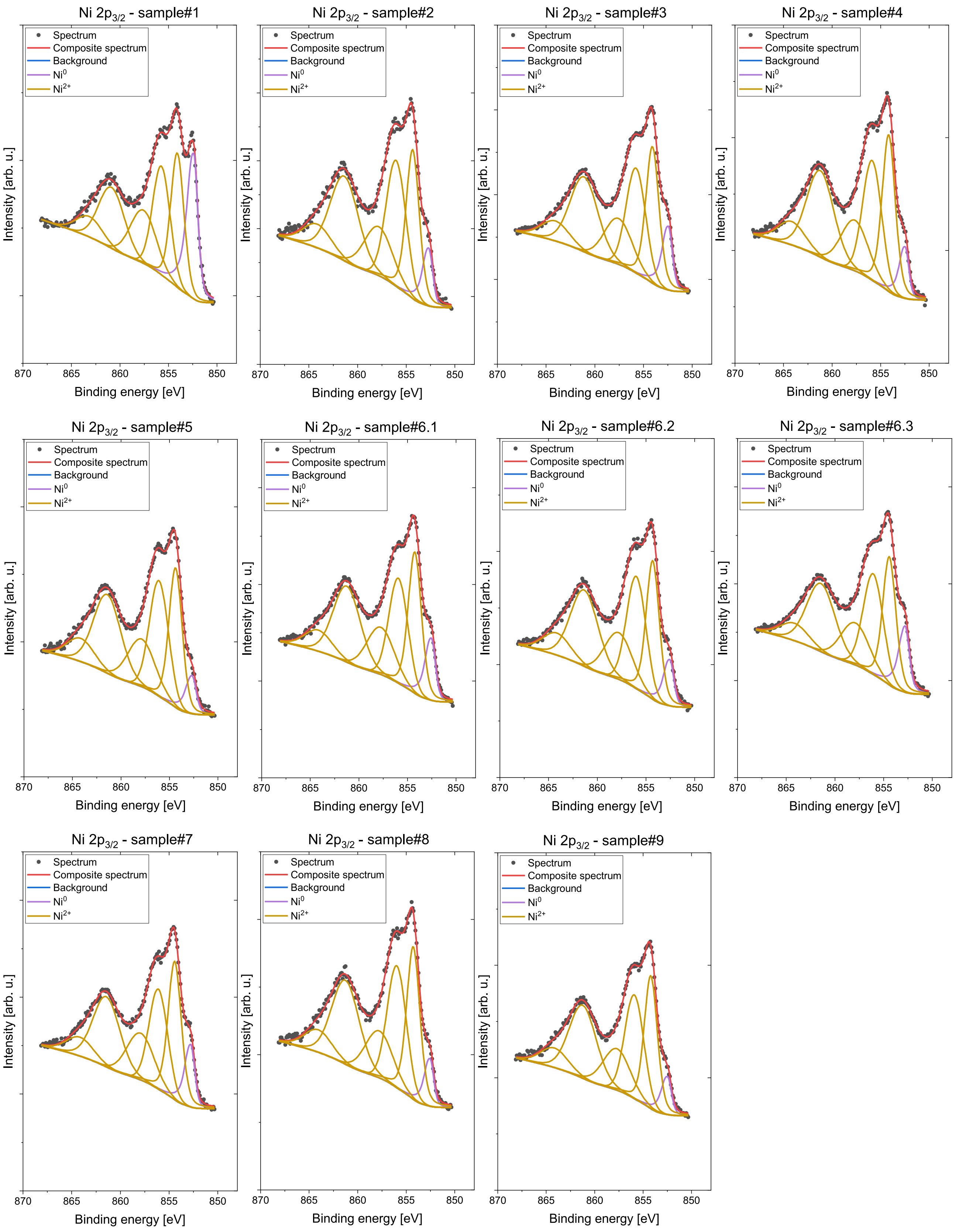

Figure S13: High resolution Ni $2p_{3/2}$ spectra of XPS measurements for all studied electrodes.

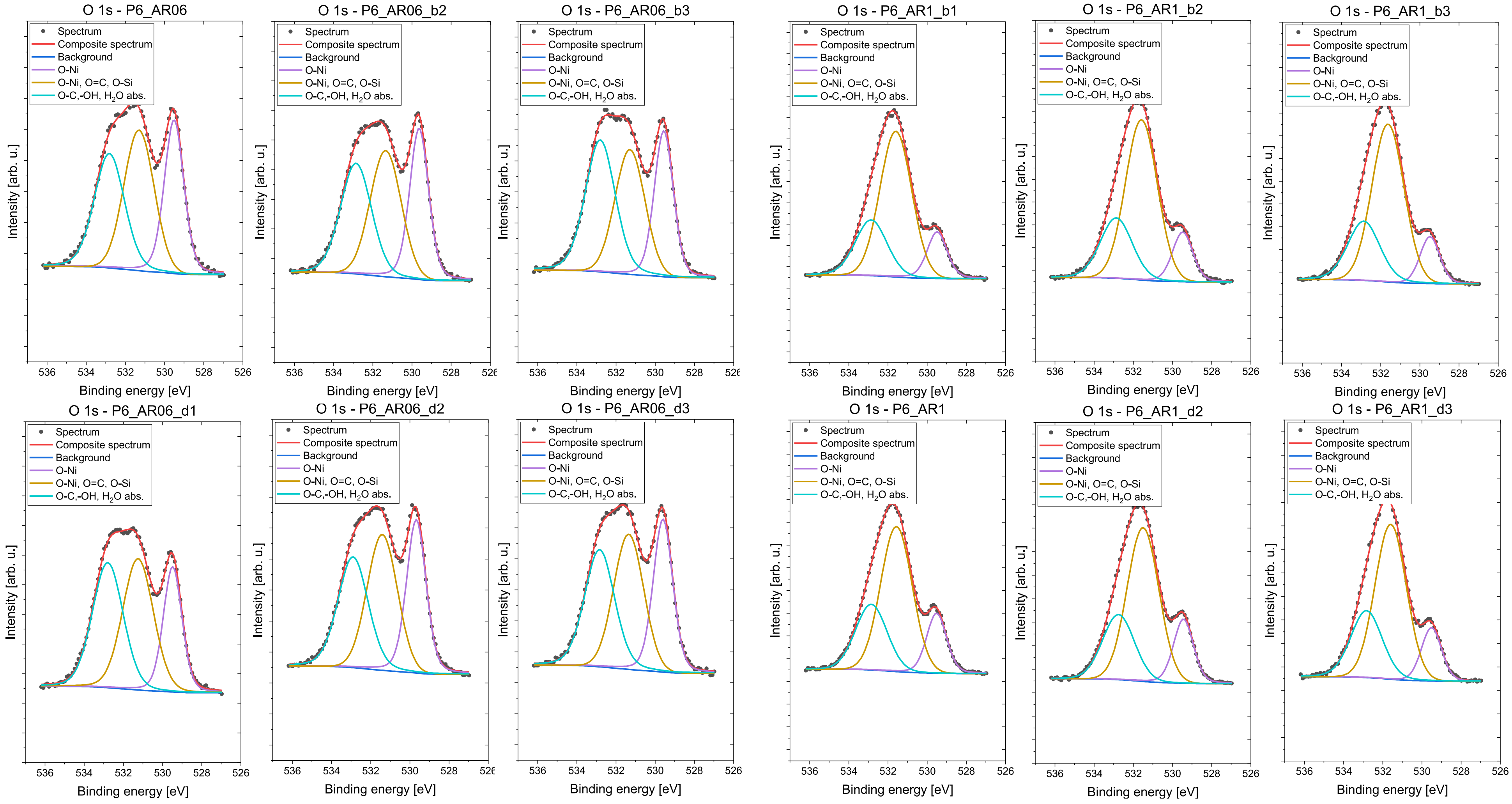

Figure S14: High resolution O 1s spectra of XPS measurements for electrode #3 and #4 with $\Lambda = 6\,\mu m$ in the high- (bright) and low intensity areas (dark).

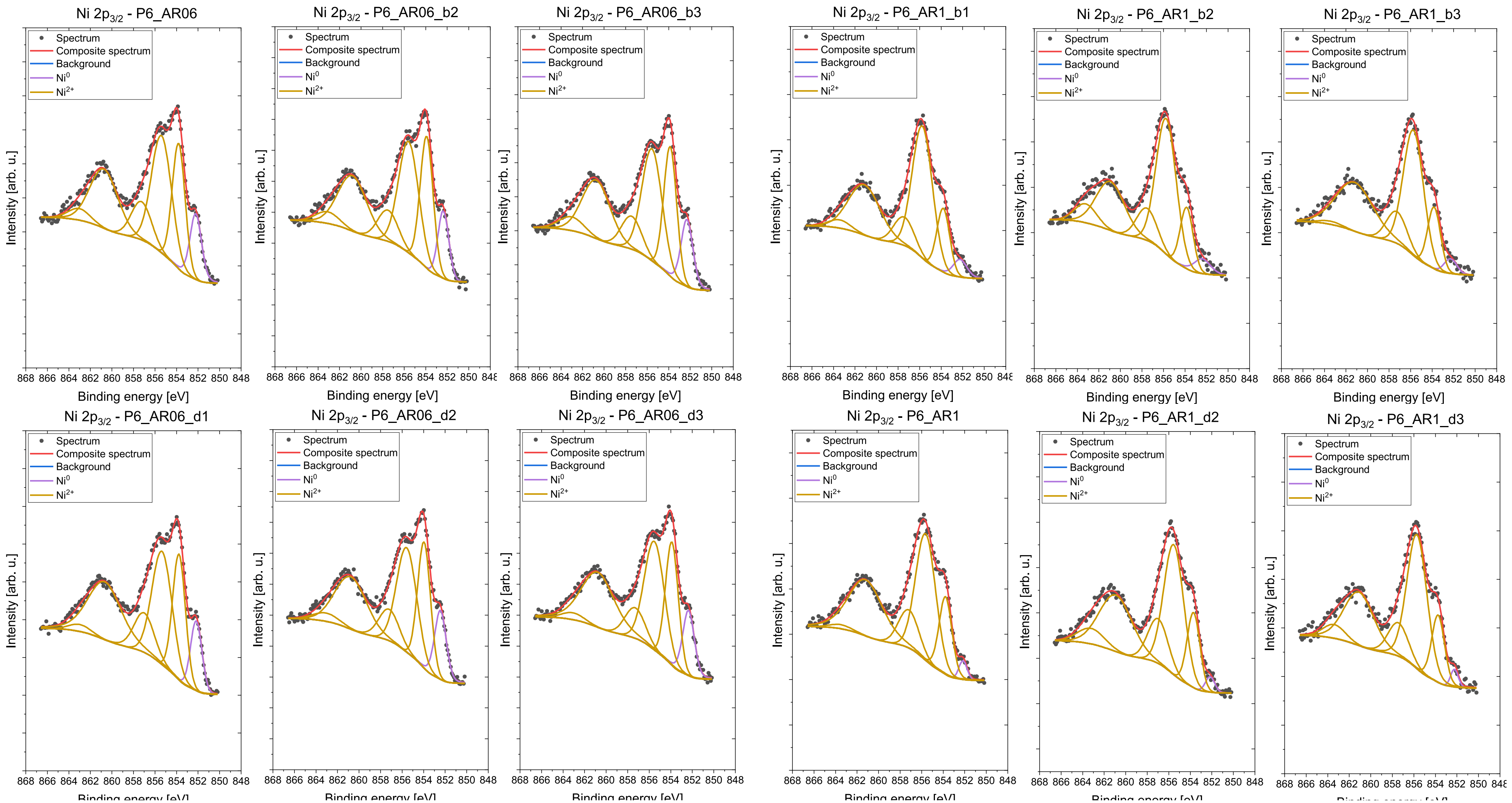

Figure S15: High resolution Ni $2p_{3/2}$ spectra of XPS measurements for electrode #3 and #4 with $\Lambda = 6\,\mu m$ in the high- (bright) and low intensity areas (dark).

Table S2: Surface composition (in atomic %) determined by fitting XPS spectra for all studied electrodes. The position of the 100 µm X-ray focus spot was chosen randomly on the electrode surface. Thus, no distinction is made between HIA and LIA.

**Element**	**C**				**N**			**O**			**P**	**S**	**Ni**	
BE (eV)	285.0	286.5	288.2	289.2	398.2	400.1	402.6	529.8	531.5	533.2	133.2	168.3	852.3	853.8
Groups, Ox. state	C-C, C-H	C-O, C-N	C=O, N-C=O N-C=N	O=C-O	N-C=N	N-C N-C=O	NH_4^+	O-Ni (latt.)	O-Ni(def.), O=C	O-C, O-H	PO_4^{3-}	SO_3^{2-}	Ni^0	Ni^{2+}
#1	25.7	7.8	3.0	2.3	0.0	2.5	0.2	10.9	17.1	8.5	0.6	0.8	4.3	16.2
#2	23.6	9.9	3.2	1.9	0.4	2.2	0.3	11.4	17.3	10.0	0.7	1.1	1.3	16.7
#3	16.2	5.6	2.3	1.1	0.5	1.4	0.0	17.3	16.3	7.1	1.5	0.4	2.7	27.6
#4	17.2	8.0	2.9	1.7	0.4	2.1	0.3	14.6	17.4	8.5	1.2	0.6	1.5	23.5
#5	30.2	7.4	1.9	0.8	0.2	1.4	0.8	12.7	17.5	5.6	0.0	1.7	1.1	18.7
#6.1	16.8	6.9	2.8	1.3	0.0	1.6	0.2	15.8	18.2	7.9	1.9	0.5	2.3	23.9
#6.2	18.5	10.9	3.9	2.3	0.2	2.1	0.3	13.7	14.9	10.8	0.0	0.9	1.4	20.3
#6.3	26.4	5.9	1.7	1.2	0.3	1.3	0.5	16.0	15.6	4.9	0.0	1.5	2.5	22.4
#7	28.6	4.9	1.1	0.5	0.2	0.6	0.4	16.7	15.6	4.3	0.0	1.4	2.3	23.4
#8	20.1	10.9	4.7	2.2	0.2	3.5	0.3	12.8	14.5	10.9	0.7	0.7	1.1	17.7
#9	19.6	10.2	4.3	2.2	0.2	3.2	0.3	12.2	16.8	10.2	0.3	0.7	1.1	18.1

S9 Models of the multiple regression analysis

For all studied responses

- Double-layer capacitance (C_{dl}),
- Onset potential (E_{on}),
- Quasi-steady state potential (E_{SS}),
- Number of nucleation sites (n_{nucl}),
- Mode (d_{m}) and median (d_{50}) value of the bubble size distributions,

response surface models were derived using multiple regression analysis for a better understanding of the influence of these factors:

- Spatial period (Λ)
- Aspect ratio (AR)
- Current density (j)

The resulting surface plots of these models are shown in Fig. S16.

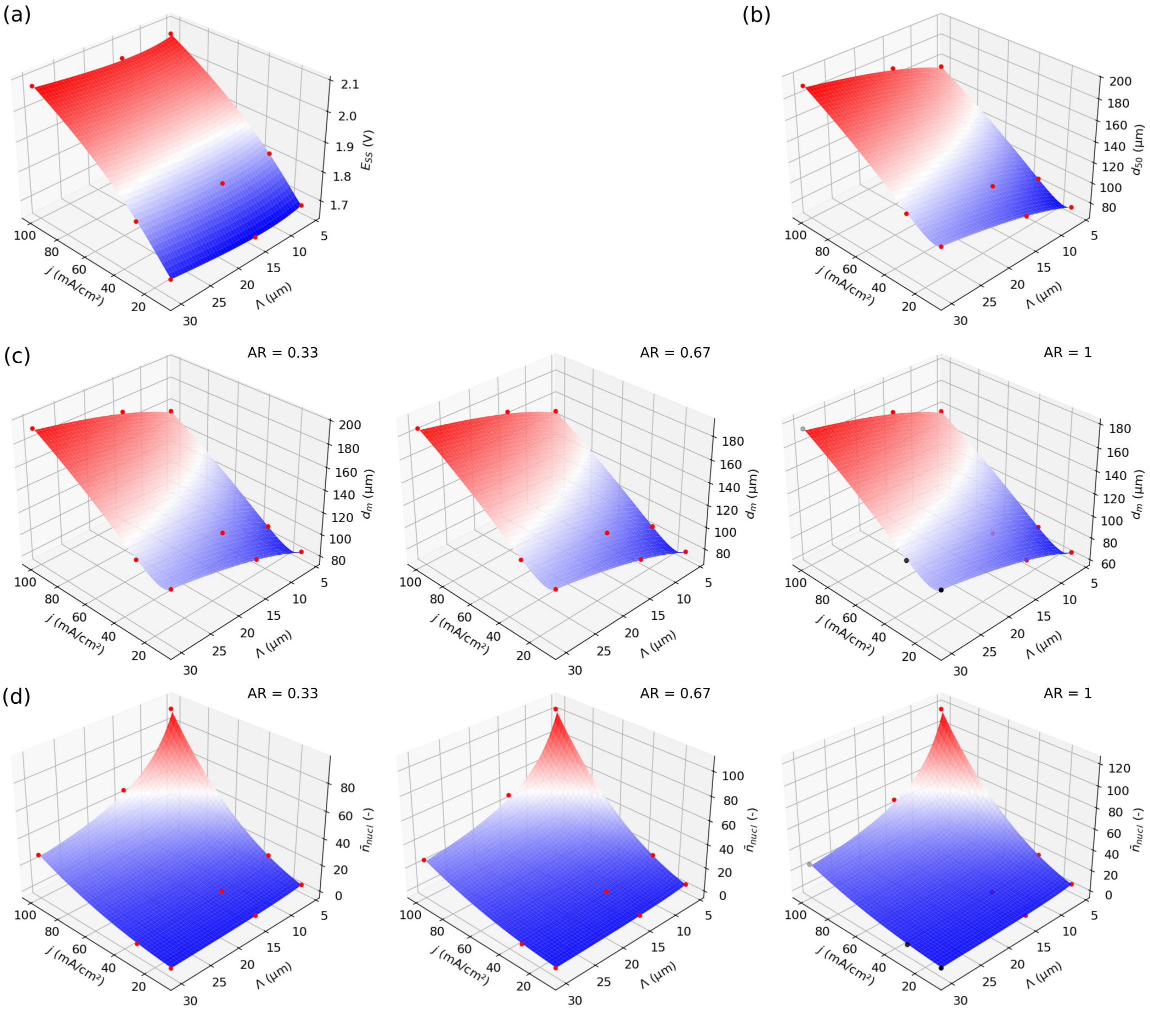

Figure S16: Surface plots of the determined models with highlighted measuring points of (a) E_{SS}, (b) d_{50}, (c) d_{m} and (d) $\bar{n}_{\mathrm{nucl}}$. Measurement points at $AR = 1$ in (c) and (d) plotted in black mark the extrapolated data points of the non-existent electrode with $\Lambda = 30\,\mu\mathrm{m}$ and $AR = 1$.

S10 Error analysis of experiments

Table S3: Measurement noise (σ) of experiments calculated with the central and reference point of DoE for double-layer capacitance (C_{dl}), onset potential (E_{on}), quasi-steady state potential (E_{SS}), number of nucleation sites (n_{nucl}) and the mode (d_{m}) and median value (d_{50}) of the bubble size distributions

Parameter	Measurement point	
	Central point	Reference point
C_{dl}	4.6991 µF	1.7194 µF
E_{on}	0.0050 V	0.0215 V
E_{SS}	0.0244 V	-
n_{nucl}	3.5447	-
d_{m}	17.0762 µm	-
d_{50}	19.3189 µm	-

S11 Data and videos

Sample data sets with raw images, electrochemical measurement data, and results can be found at 10.14278/rodare.3064. Due to the size of the complete data, the remaining image data can be made available upon request.

The provided characteristic videos in the dataset are named after following scheme:

Perspective_Electrode_CurrentDensity → *E.g.: Sideview_#1_NSE_100mAcm-2*

Two supporting videos are provided. The video named S11_1 is showing exemplary videos of the wetting behavior from a non- and laser-structured electrode and S11_2 is showing bubble coalescence on the electrode surface during the bubble growth.

References

[1] G. Beamson and D. Briggs, *J. Chem. Educ.*, 1993, **70**, A25.

[2] P. G. Rouxhet and M. J. Genet, *Surf. Interface Anal.*, 2011, **43**, 1453–1470.

[3] N. Liu, T. Li, Z. Zhao, J. Liu, X. Luo, X. Yuan, K. Luo, J. He, D. Yu and Y. Zhao, *ACS Omega*, 2020, **5**, 12557–12567.

[4] M. J. Genet, C. C. Dupont-Gillain and P. G. Rouxhet, in *Medical applications of colloids*, ed. E. Matijevic, Springer, 2008, vol. 177, pp. 177–307.

[5] C. D. Wagner, D. E. Passoja, H. F. Hillery, T. G. Kinisky, H. A. Six, W. T. Jansen and J. A. Taylor, *J. Vac. Sci. Technol.*, 1982, **21**, 933–944.

[6] A. Wagner, A. Naumkin, A. Kraut-Vass, J. Allison, C. Powell and J. Rumble, 2003, `http:/srdata.nist.gov/xps/`.

[7] M. Fantauzzi, B. Elsener, D. Atzei, A. Rigoldi and A. Rossi, *RSC Adv.*, 2015, **5**, 75953–75963.

[8] M. C. Biesinger, B. P. Payne, L. W. M. Lau, A. Gerson and R. S. C. Smart, *Surf. Interface Anal.*, 2009, **41**, 324–332.

[9] M. C. Biesinger, B. P. Payne, A. P. Grosvenor, L. W. M. Lau, A. R. Gerson and R. S. C. Smart, *Appl. Surf. Sci.*, 2011, **257**, 2717–2730.

[10] M. C. Biesinger, L. W. M. Lau, A. R. Gerson and R. S. C. Smart, *Phys. Chem. Chem. Phys.*, 2012, **14**, 2434–2442.
